# Distribution of Cement Content Across Wall Thickness of Spun-Cast Concrete Poles and Its Implications for Their Durability

**DOI:** 10.3390/ma19173718

**Published:** 2026-08-31

**Authors:** Jarosław Michałek

**Affiliations:** Faculty of Civil Engineering, Wrocław University of Science and Technology, 27 Wybrzeże Stanisława Wyspiańskiego st., 50-370 Wrocław, Poland; jaroslaw.michalek@pwr.edu.pl

**Keywords:** prestressed poles and towers, spun concrete, durability, morphology of concrete, cement

## Abstract

The durability of prestressed spun-cast concrete poles is typically assessed on the basis of the nominal mix design parameters and the assumption of material homogeneity. However, the centrifugal spinning of fresh concrete mix results in an uneven distribution of its components throughout the wall thickness, which may affect the actual cement content in individual layers and consequently, the durability. As part of this study, the distribution of cement content in spun concrete poles was analysed using an experimental approach based on the analysis of hardened concrete composition. Samples were taken from various locations along the height of the pole and divided into layers across wall thickness. Cement content, aggregate distribution, porosity, and water absorption were determined using a combination of chemical and physical methods. The results indicate that although the average cement content meets the design requirements (minimum amount of cement exceeds 300 kg/m^3^), there are significant local variations, particularly in the inner and outer layers of the cross section. The inner layer is characterized by increased cement content and porosity, while the outer layer is characterized by a higher coarse aggregate content and reduced cement content. It was also observed that the spinning program used in the production of the poles resulted in a homogeneous concrete structure at the top of the pole, where the spinning radius is smallest, with no signs of delamination. However, delamination of the concrete structure was observed at lower sections of the pole. These results highlight the limitations of assuming homogeneous material properties in durability design and suggest that the actual performance of spun concrete elements may deviate from predictions based on the standards. The results have direct implications for assessing the durability of spun concrete poles used in exposure classes such as XC4 and XD1.

## 1. Introduction

Spun-cast concrete elements, such as prestressed poles and towers, are widely used in construction due to their high strength, durability and efficient material utilization. Their performance in service is typically assessed using standardized parameters such as minimum cement content, water-cement ratio, compressive strength, and concrete cover thickness. The parameters are defined assuming material homogeneity throughout the cross section.

The durability requirements for spun concrete poles and towers in standards [1,2,3] are for the expected service life of 50 years. According to standard [4], when designing spun concrete poles and towers, one can assume a design lifespan of 30 years (Class A) or 50 years (Class B).

As regards durability, it should be noted that the first electric power and light poles made of spun concrete were manufactured in Germany at the beginning of the 20th century [5,6]. Later, this advanced technology was used in other countries (e.g., France and Belgium) for the production of, among other things, poles and pressure pipes. After World War II, spun concrete poles began to be mass-produced in the former Soviet Union [7]. In the 1960s and 1970s, the production of spun concrete poles significantly expanded in Western and Eastern Europe [8], as well as in the USA [9,10,11] and Canada. In northern and western Poland, spun concrete electric power and light poles manufactured back in the 1930s can still be found in operation. The oldest power poles manufactured in Poland using an original Polish technology have been in successful operation for over thirty years [12].

The expected environmental conditions, among other factors, must be taken into account in order to ensure adequate durability of a structure. The above-ground portion of prestressed spun concrete poles and towers is subject to the action of class XC4 environment (cyclically wet and dry surfaces exposed to corrosion caused by concrete carbonation). The above-ground portion of spun power and light poles located along roads and streets, being in contact with the ground in the de-icing agent splash zone, is additionally subject to the action of environment XD1 (moderately damp concrete surfaces exposed to airborne chlorides) and XF2 (moderately water-saturated vertical concrete surfaces exposed to freezing/thawing and airborne de-icing agents). The lower portion of power poles embedded in the ground [13] may exceptionally be exposed to environment XA1 (the aggressive chemical environment of soils and groundwater).

Standard [2] specifies requirements for ordinary concrete for different environmental exposure classes. For exposure class XC4, the maximum *w*/*c* ratio is 0.50, the minimum cement content is 300 kg/m^3^, and the minimum concrete strength class is C30/37. For exposure classes XD1 and XA1, the maximum *w*/*c* ratio increases to 0.55, while the minimum cement content and minimum concrete strength class remain unchanged. For exposure class XF2, the maximum *w*/*c* ratio is 0.55, the minimum cement content is 300 kg/m^3^, and the minimum concrete strength class is C25/30.

Standard codes for prestressed spun concrete elements do not specify frost resistance requirements for the XF2 environment. Standard codes [2] merely state that aggregate with appropriate frost resistance and a concrete mix with a minimum air content of 4.0% need to be used. Because of the necessary durability of spun elements in freezing/thawing conditions, standard [14] suggests that the concrete water absorption should not exceed 5.0%.

In the case of chemically contaminated soil or water (exposure class XA1) whose parameters exceed the limit values of standard [2], special tests should be carried out to determine the appropriate operating conditions for a given precast concrete element and, if need be, the surface concrete should be protected against chemical attack. For XA1 environments, there is no mandatory need to coat the butt end of spun concrete power poles with insulating agents at the production stage of the precast elements or during their assembly on site [13].

Because of the required high strength of spun concrete, pure Portland clinker cement of class no lower than 42.5 is used for the production of spun concrete elements. The cements used should have increased resistance to sulphates. The tricalcium aluminate (C3A) content should not exceed 6.0%. Besides resistance to sulphate aggressiveness, such cements are characterized by high frost resistance and reduced calorific value [15]. Additionally, to eliminate “aggregate-alkali” corrosion, it is recommended to use cements with a low alkali content. Low-alkaline cement should contain no more than 0.6% alkali oxides, expressed as Na_2_O [7]. 

Aggregates for the production of spun concrete elements should exhibit high strength and a high modulus of elasticity, good adhesion of the cement paste to the aggregate particles, and a proper particle size distribution. These requirements are best met by crushed mineral aggregates. The basic rock raw materials for crushed aggregates are igneous rocks (most often granite and basalt, less frequently porphyry and diabase) or Devonian dolomites [16,17,18,19]. The aggregate class cannot be lower than the required concrete class.

Protection of the steel in prestressed spun concrete elements is ensured by an appropriate concrete cover thickness. Minimum concrete covers c_min_ for non-prestressed and prestressed reinforcement for a 50-year design service life span (design class S4) are given in code [1]. Prestressed spun concrete poles and towers are manufactured in precast plants where, in accordance with product standard [4], factory production control must be in place (including concrete and concrete cover thickness control [20], whereby the execution class is lowered to S3). This, combined with the achieved concrete strength class ≥ C40/50 (in towers ≥ C50/60), means that the execution class can be lowered to S2 [1]. Consequently, a 10 mm reduction in the minimum concrete cover is obtained (Table 1).

Table A.1 of standard [21] specifies the minimum concrete covers for non-prestressed and prestressed reinforcement, assuming that quality control is applied during the production of precast elements as part of factory production control. The concrete covers specified in standard [21] (Table 1) are identical to the value resulting from the provisions of standard [1]. The alternative conditions specified in Annex A.2 of standard [21] extend the conditions relating to the minimum concrete cover. The alternative conditions indicate that if class ≥ C40/50 concrete with water absorption less than 5.5% is used in a precast element, the concrete cover thickness specified in Table A.1 [21] may be reduced by 5 mm (Table 1). Whereas, if class ≥ C50/60 concrete with water absorption less than 4.5% is used in the precast element, the concrete cover thickness specified in Table A.1 [21] may be reduced by 10 mm (Table 1). Thus, the provisions of standard [21] regarding the minimum reinforcement cover significantly reduce c_min_ in relation to the values given in standard [1] for the 50-year service life span of the structure.

Table B.1 of standard [4] specifies minimum conventional concrete reinforcement bar covers for spun concrete poles and towers for the 30-year service life span. These values are 5 mm lower than the ones given in standards [1,21], which is due to the inclusion of factory production control [20]. The omission of prestressed concrete elements in standard [4] seems to be incorrect, as the prestressing tendon concrete cover thickness should be 10 mm greater than the values given in Table B.1 [4] (the author’s proposal is given in Table 1).

In the provisions of Annex B of standard [4] there is additional information (which is inconsistent with the provisions of standard [21]) that for concrete with compressive strength class ≥ C50/60 and water absorption less than 3.5% the cover may be reduced by 5 mm, but in no case should the thickness of the concrete cover be less than 10 mm for stirrups or spirals and not less than 15 mm for longitudinal bars.

In the case of spun concrete elements made of class ≥ C40/50 concrete with water absorption below 5.5% manufactured in Poland, the minimum cover thickness (mainly for exposure class XC4) for the 50-year service life span assumed in accordance with the alternative conditions of point A.2 of standard [21] amounts to c_min_ = 25 mm for prestressing steel and to c_min_ = 15 mm for plain steel (including helical reinforcement). Reduction of the minimum pre-tensioned tendon cover thickness to c_min_ = 20 mm in spun concrete elements with the design life span of 50 years is possible if the manufacturer manages to make spun concrete of class ≥ C50/60 with water absorption below 4.5% (the alternative conditions of point A.2 of standard [21]).

**Figure 1:**
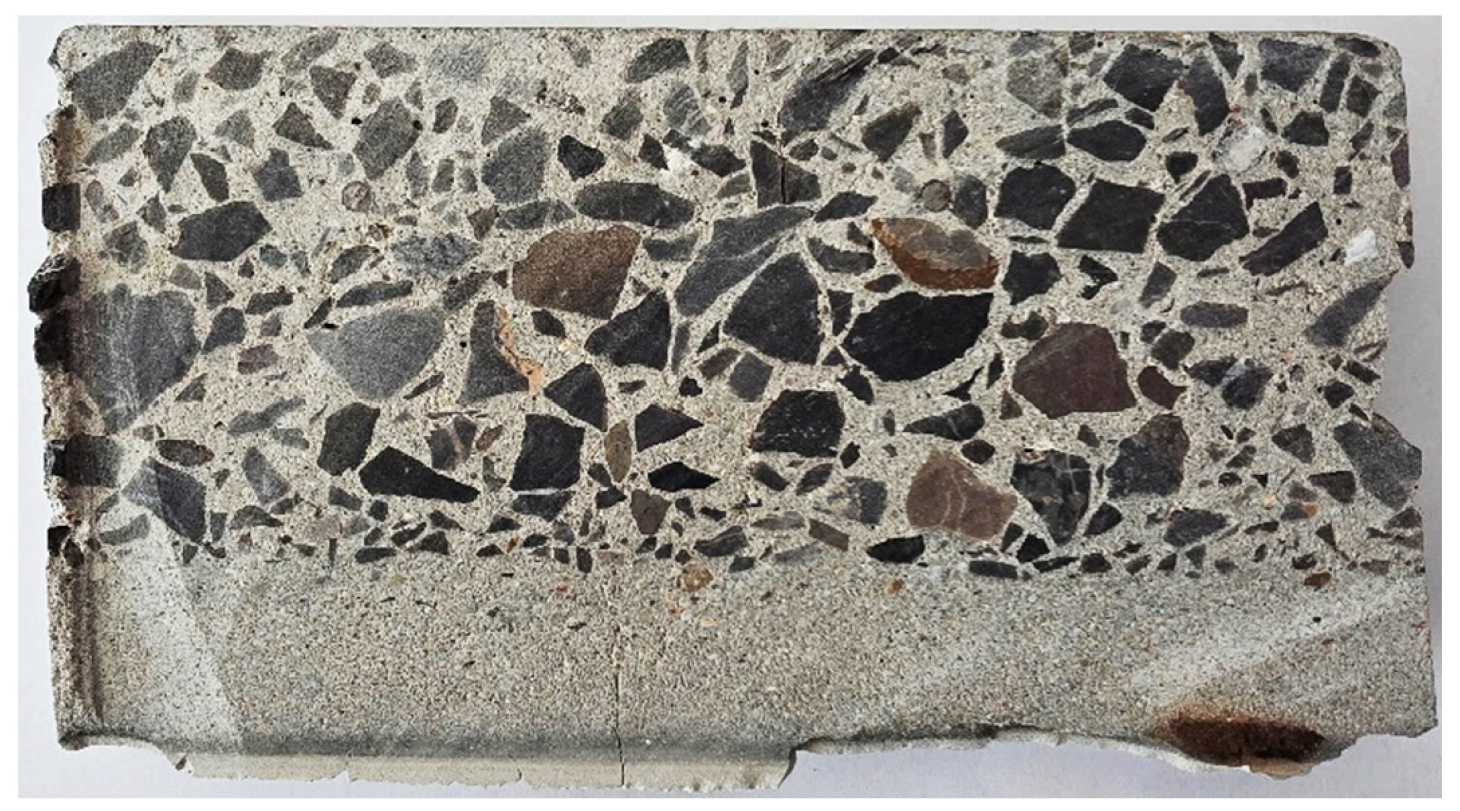
Layered structure of spun concrete—cross section through wall thickness.

Concrete spin casting consists of forming and compacting a concrete mix under the action of a normal (radial) force generated during the rapid rotation of the mould around its longitudinal axis at a speed of 500–700 rpm. Because of its peculiar nature, this method is applicable only to elements with an annular cross section. Unlike the structure of vibrated concrete, the structure of concrete produced by spin casting is not homogeneous throughout the element wall thickness, but rather heterogeneous and layered [22,23,24,25,26,27,28,29,30,31,32,33,34,35,36,37]. During concrete spin casting, heavier constituents (coarse particles) collect in the cross section’s outer zone, while lighter components (paste) collect in its inner zone (Figure 1). As a result, the outer zone can achieve a high constituent content and, after setting, high strength and high resistance to chemical and mechanical impacts. Whereas the inner layer will consist of highly compacted, very cohesive cement paste and, after setting, can achieve high impermeability.

The layering of the cross-section depends on many factors, including technological factors such as spinning time, rotational speed, and the spinning program; geometric factors such as mold diameter; and material factors such as the initial cement content and concrete mix consistency.

One of the first researchers to describe the structure of spun concrete used for pipe production was Marquardt E. [23]. On the basis of his observations, he concluded that the greater the difference in the specific gravity of the particular components of the concrete mix, the faster and deeper its fractionation. He recommended using well-graded aggregate mixtures with similar petrographic properties and the maximum particle diameter of 15 mm. He also discussed the variation in cement content across the wall thickness of spun concrete pipes. He demonstrated that cement is relatively uniformly distributed over approximately 83% of the wall thickness, with an increase in its content occurring only in the 2–5 mm thick inner layer of the cross section. To reduce cross-section stratification, he proposed the use of layered spin casting at lower mould rotational speeds.

Akhvyerdov [24] confirmed in his analyses and studies that the effect of centrifugal force during the spinning of a concrete mix increases with increasing distance from the axis of rotation and the specific gravity of the particles. As a result, larger particles move towards the element’s outer surface, while finer particles collect closer to its inner surface. Hence, spun concrete differs from vibrated concrete in the inhomogeneous distribution of aggregate particles across the wall thickness of the product. For a properly selected concrete mix composition, a 0.5–2.0 mm thick layer of slurry forms on the element’s inner surface, followed by a cement paste layer, fine concrete, and finally concrete with an ordinary structure. Along with layering, cement distribution in the product wall cross section also changes. This inhomogeneity causes a decrease in the strength of spun concrete in comparison with a material with the uniform distribution of aggregate particles throughout the cross section.

Microscopic examinations carried out by Dilger, Ghali [25,26] and Rao [27] clearly revealed that coarse and fine aggregates are fractionated across the cross-section wall thickness. Coarse aggregates were concentrated mainly in the outer zone (75–80%), amounting to merely about 25% at the inner surface. Moreover, a higher air content was observed near the inner surface than at the outer surface. The mortar was distributed relatively evenly, but its amount increased towards the inner surface. The lowest mortar content was noted at the outer surface (15–20%), while near the inner surface it increased to 40–50%. In the inner zone, a distinct layer of 6–7 mm thickness formed, which underwent cracking due to differential shrinkage between the inner layer and the outer layer.

In study [28], Dilger and Rao drew attention to the importance of centrifugal spinning parameters, such as speed and time. High rotational speed ensures good compaction of the concrete mix, but at the same time contributes to aggregate segregation. The study showed that using crushed limestone (dolomite) aggregate, one can obtain higher concrete strength since the bond between this type of aggregate and the cement paste is stronger than in the case of gravel concrete. Mix segregation during centrifugal spinning can be significantly reduced by reducing the fine components fraction, i.e., through a proper fine-to-coarse aggregate ratio. The optimal sand content was found to amount to 25% of the total aggregate mass for gravel concrete and to 22.5% for limestone concrete.

Adesiyun et al. [29,30,31] used computer image analysis to quantitatively and qualitatively describe the structure of spun concrete. For detailed analysis, the sample wall cross-section was divided into 20 strips, 2.25–3.00 mm wide depending on the wall thickness. Tests were conducted for various combinations of parameters, such as sand equivalent (25–50%), cement content (410–530 kg/m^3^), plasticizer content (0–2%), spinning speed (400–700 rpm), and spinning time (5–11 min). Thanks to image analysis, a visual representation of the distribution of aggregate, paste, and air voids across the wall thickness of the samples was obtained. It was shown that aggregate content decreased from the outer zone towards the inner zone, while the paste content varied inversely, reaching almost 100% in the inner zone. Air content was higher in the inner layers than in the outer layers. Compositional segregation was observed in all the samples. The lowest fractionation was observed for the sample with the lowest sand equivalent (25%), the lowest spinning speed (400 rpm), and the shortest process time (5 min).

Kuranovas and Kvedaras in [32] presented concrete spinning as a four-phase process. The first phase involves feeding the concrete mix into the mould and evenly distributing it along the mould length at a low rotational speed of the centrifuge. As the mould rotational speed increases, the process transitions to the second phase, in which the centrifugal force compresses the concrete mix and forms a layered concrete cross-section wall. In the third phase, the rotational speed of the mould is further increased, which leads to concrete mix compaction, cross-section wall thickness stabilization, and squeezing some of the mixing water into the inside. The fourth phase occurs at the maximum rotational speed of the mould and involves further concrete mix compaction and excess mixing water removal. In this phase, the element wall reaches the designed thickness.

Völgyi et al. [33,34] found that the segregation of concrete mix during centrifugal spinning depends largely on the excess cement paste and the degree of compaction. Properties such as strength, porosity, hardness, and material composition vary across the thickness of the spun concrete wall due to the segregation of components. This phenomenon can be limited by reducing the amount of paste and using the optimal compaction effort. A relationship between concrete cohesion and the recommended compaction effort was also proposed. Furthermore, a formula for determining the centrifuge operating parameters, i.e., time and rotational speed, to obtain optimal compressive strength parameters of the samples was developed.

By conducting tests on cylindrical spun concrete specimens with an outer diameter of 65 mm and a height of 80 mm, Dedukh, Schsuzkiy and Kuzmenko [35] demonstrated that spun concrete is characterized by the segregation of components across the cross-section wall thickness, which leads to differences in the physical and mechanical properties of the material. The difference in average density between the outer layer and the inner layer ranged from 4 to 6%, while the porosity of the inner layers was more than 18% higher than that of the outer layers. The inner layer contained a larger amount of water and cement. The cement content in the inner layer amounted to approximately 510 kg/m^3^, while in the outer layer it was about 460 kg/m^3^, with the initial cement content in the concrete mix amounting to 500 kg/m^3^.

Michałek, Pachnicz and Sobótka in [36] presented a test methodology combining three different test methods: nanoindentation, X-ray computed microtomography (mCT) and two-dimensional optical scanning. The methods were used to describe selected features of the internal microstructure of spun concrete, such as the distribution of concrete components, the variability of the mortar’s mechanical parameters across cross-section wall thickness, and the spatial air void distribution. The tests were conducted on a sample made from a mixture used for the production of spun power poles in a prefabrication plant in Poland. The obtained results provided not only qualitative information but also a quantitative description of the distribution of components across the wall thickness of the spun concrete element. It was demonstrated, among other things, that under the adopted spin casting parameters, cement is distributed relatively evenly across the wall thickness, and its content increases only in the inner layer of the cross section.

In study [37], Michałek and Sobótka, using imaging techniques and suitable image analysis, showed that the cement distribution is relatively constant as a function of spun concrete sample wall thickness, while an increase in its content was observed only in the approximately 2 mm thick inner layer of the cross section. The results of the investigations confirmed the effectiveness of the conventional test method [38], and they are almost entirely consistent with the conclusions presented earlier by Marquardt [23].

Kliukas, Jaras and Lukoševičienė [7] found that each phase of the concrete spinning process is characterized by a specific rotational speed depending on the dimensions of the element and the time needed to reach the desired speed. According to [24], the duration of the first phase should be 3–4 min, the second and third phases 1–2 min, and the fourth phase 10–15 min. It should be noted that the time of the centrifugal spinning of the element in a steel mould also depends on the initial *w*/*c* ratio of the concrete mix and on the pressing pressure generated by the centrifugal force, which depends on the rotational speed and the dimensions, especially the diameter of the product.

The research done so far has confirmed the occurrence of material segregation across the cross-section wall thickness and its impact on the aggregate distribution, porosity, and microstructural properties of spun concrete. In particular, an increased cement paste concentration and higher porosity are found in the inner layers, while the outer layers are typically more compact and contain a larger coarse aggregate fraction. Despite these findings, relatively little attention has been paid to the quantitative assessment of cement content distribution across the cross-section and its impact on material durability. Despite these findings, relatively little attention has been paid to the quantitative assessment of cement-content distribution across the element cross-section and its implications for compliance with standard durability requirements.

From the durability perspective, this issue is particularly important. The outer zone of spun concrete elements, constituting the concrete cover, is directly exposed to environmental impacts such as carbonation, chloride penetration, and freeze-thaw cycles. The resistance of this zone depends largely on its composition and microstructure. If the local cement content in the outer layers deviates from the value assumed in the concrete mix design, the actual durability of the element may differ from the durability predicted on the basis of the standard requirements.

Current design approaches and standards define durability criteria based on global concrete mix parameters, without directly considering the inhomogeneous distribution of components resulting from centrifugal spinning. This raises the question of whether the assumption of material homogeneity is justified for spun concrete elements and whether local compositional differences may affect the long-term performance of such elements.

The aim of this study was to investigate cement content distribution and selected material parameters in the cross section of the wall of prestressed spun concrete poles. This was done through an experimental analysis of hardened concrete samples collected from various heights of a full-size spun concrete element. Particular attention was paid to cement content variation, aggregate distribution, porosity, and water absorption. Particular attention was paid to variations in cement content, aggregate distribution, porosity, and water absorption in various cross-sections located at the height of the element.

The results obtained may provide further insight into the internal structure of spun concrete and help evaluate the validity of current assumptions regarding the durability of this material. The findings of this study may contribute to the development of improved methods for assessing the long-term performance of spun concrete elements, as well as to more effective design approaches for structures intended for service under aggressive environmental conditions. The study also provides a basis for a more in-depth analysis of the concrete spinning technology used at the precast plant and the associated cement consumption.

## 2. Preparation of Test Samples

A prestressed concrete electric traction pole of type ETG-1, manufactured at a precast concrete plant, was used as the test specimen. However, because the pole was manufactured as part of the standard production process, the parameters affecting the structure of the spun concrete could not be varied independently. The results should therefore be regarded as a case study rather than as a systematic investigation of the effects of individual parameters on the strength and deformability of spun concrete.

The ETG-1 pole is one of four variants of the ETG series of partially prestressed traction poles with a standard length of 8.2 m, differing in the function performed in the tension section. ETG traction poles at their base are equipped with a steel head enabling attachment to reinforced concrete pile foundations using bolted connections [39,40,41]. In the traction pole trade symbols, the letter part (ETG) indicates the type of pole—an electric power traction pole with a steel base plate, and the digital part after the dash indicates the load-bearing function of the pole in the tension section (1—a through pole, 2—a cross and central anchoring pole, 3 and 4—an anchor pole). Rated apex force P_k_ is defined as the equivalent characteristic force applied at a distance of 0.2 m from the top of the pole.

The ETG-1 pole is a prefabricated partially prestressed element with a lengthwise variable annular cross section and a longitudinal truncated cone shape. The shape of its cross section results from the adopted technology of manufacturing by concrete centrifugation in steel moulds which cannot be opened longitudinally [42]. The outer diameter of the ETG-1 pole increases from d_w_ = 177 mm at the top to d_p_ = d_w_ + L × 15 mm = 177 + 8.2 × 15 = 300 mm at the bottom with a constant taper of 15 mm/m. According to the design, the wall thickness of the annular cross section at the top should be 60 mm and at the steel head—75 mm.

The ETG-1 pole was longitudinally reinforced with prestressing tendons (active reinforcement in the form of Y1670C wires with a diameter ≥ 7.5 mm) and plain carbon steel bars (passive reinforcement in the form of ribbed steel bars with a characteristic yield strength of f_yk_ = 500 MPa and lengths adjusted to the bending moment envelope M_Ed_), distributed evenly around the circumference. The ≥7.5 mm profiled wires are upset-forged at one end and anchored in the steel head [39], while at the other end (at the top of the pole) they are bond-anchored using Gifford jaw clamps. The pole’s transverse reinforcement (helical reinforcement) was made of a smooth wire with a diameter of 3.5 mm and a characteristic yield strength of f_yk_ ≥ 500 MPa. According to the manufacturer, the ETG-1 pole was made of class C40/50 spun concrete. The nominal cover thickness for a 50-year service life is c_nom_ = 25^+10^_-5_ mm for prestressing steel and plain longitudinal steel, and c_nom_ = 20^+10^_-5_ mm for plain steel (helical reinforcement). The concrete mix design used in the production of the poles was obtained from the manufacturer (Table 2).

The use of Devonian dolomite for the poles had been preceded by a thorough analysis by the manufacturer [16,17,18,19]. The ETG-1 column was manufactured using a roller centrifuge compacting the concrete mix for approximately 460 s according to the following program:

- acceleration to 300 rpm—30 s,
- compaction at 300 rpm—90 s,
- acceleration to 650 rpm—90 s,
- compaction at 650 rpm—250 s.

The spinning program for the concrete mix described here was established at the precast plant to achieve the required fresh-concrete properties as quickly as possible while ensuring that the ring cross-section remained undamaged during spinning and during transport of the mold containing the formed element to the heat treatment chamber.

**Figure 2:**
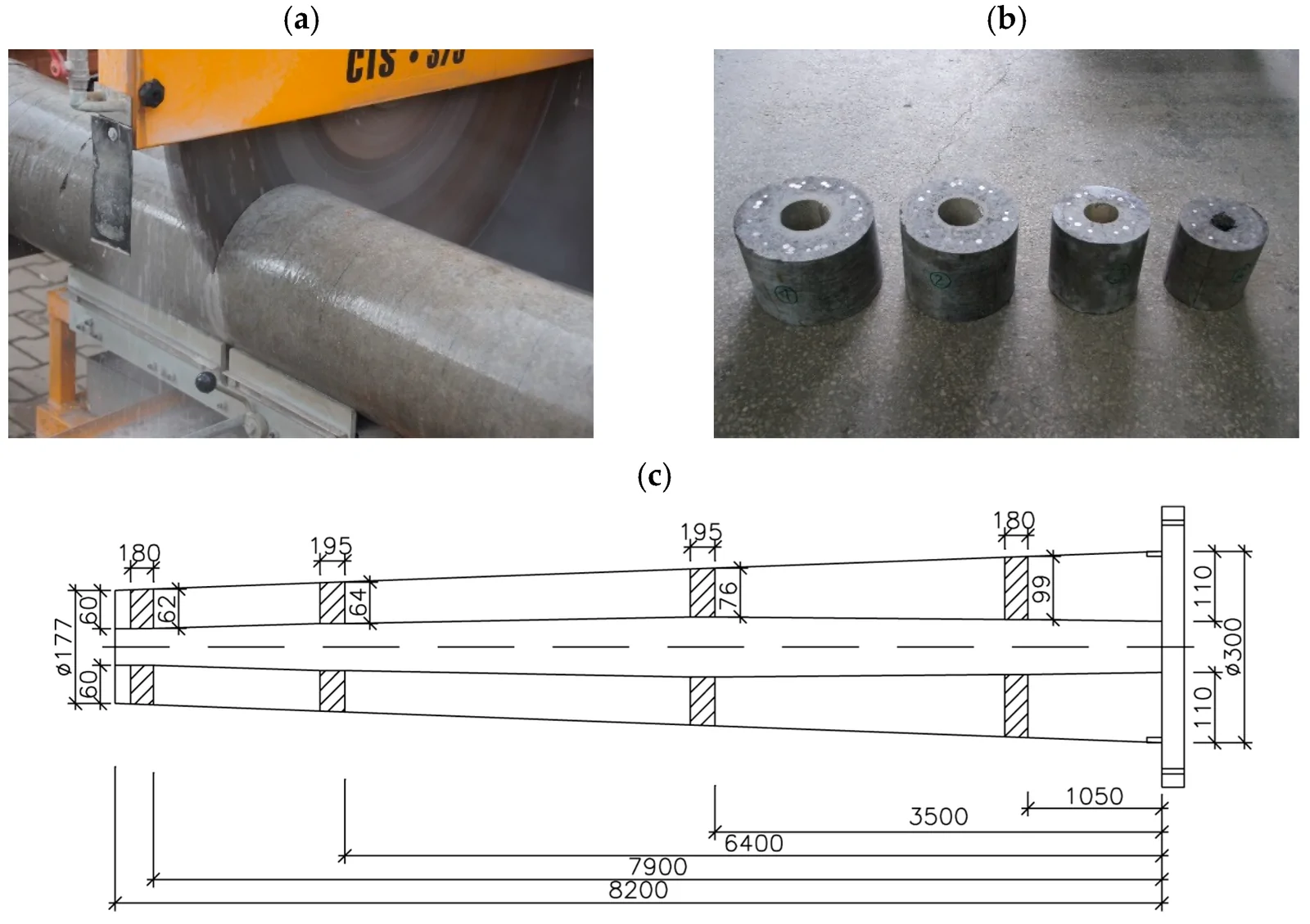
Preparation of test samples: (**a**) cutting of ETG-1 pole, (**b**) cut-off pole sections, (**c**) sketch showing distribution of sample taking places along pole length (figure was not drawn to scale, all units: mm).

For laboratory tests, four approximately 180–195 mm long sections were cut off from the ETG-1 pole (Figure 2a) from different locations along its length, whereby samples in the shape of a hollow truncated cone (Figure 2b) were obtained. The test samples were taken (Figure 2c), counting from the pole base and taking into account the length (8.2 m) of the pole’s concrete part, at a distance of 1.05 m (sample A), 3.50 m (sample B), 6.40 m (sample C) and 7.90 m (sample D), respectively. The wall thickness of the cut-off pole sections amounted to about 99 mm for sample A, 76 mm for sample B, 64 mm for sample C, and 62 mm for sample D.

**Figure 3:**
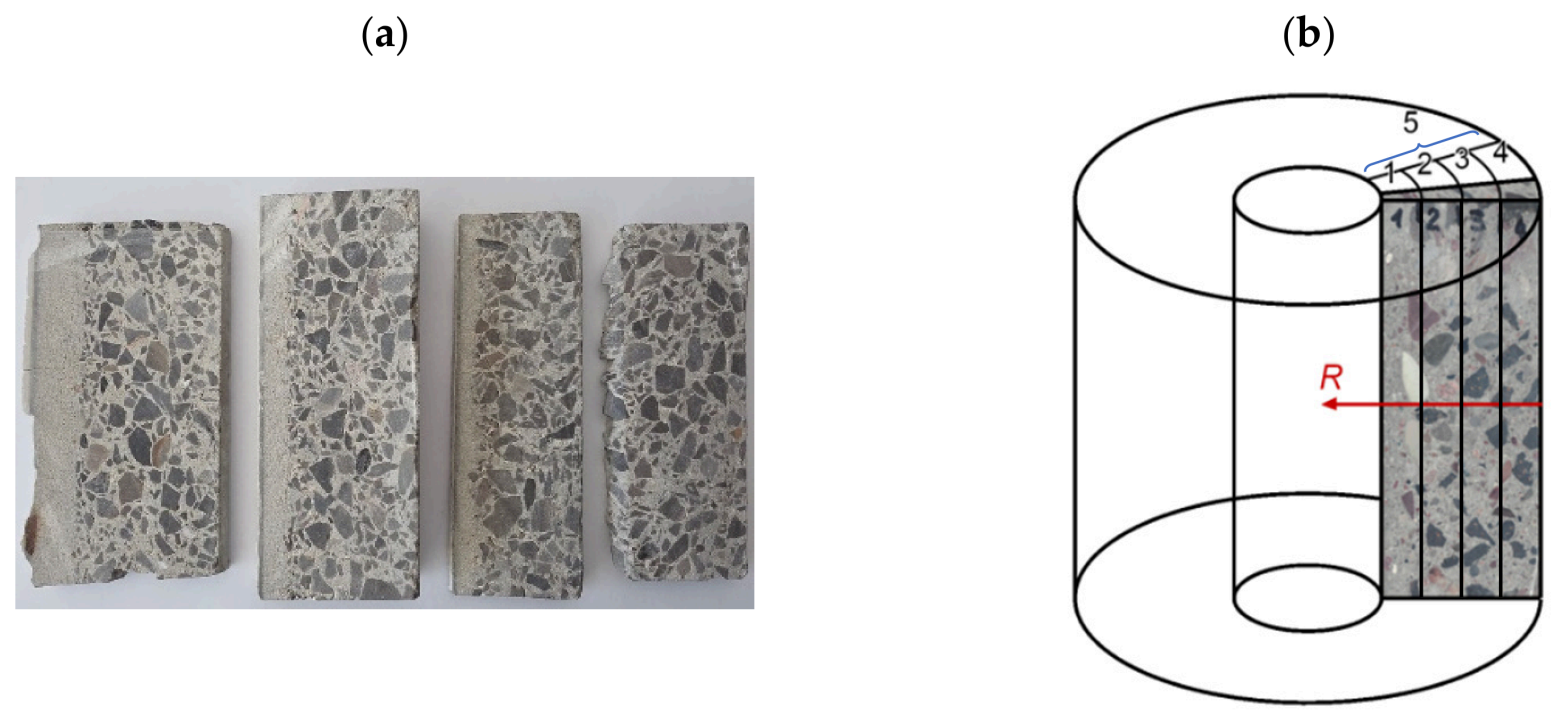
Samples for material tests: (**a**) concrete slices for laboratory tests (from left, sample A followed by samples B, C and D), (**b**) designation of samples 1 ÷ 4 and 5 for laboratory tests.

The cut-off pole sections were then cut along the generating line into approximately 15–30 mm thick slices (Figure 3a), avoiding the prestressing wires and the conventional reinforcement. Because of the layered structure of spun concrete, samples were prepared for further laboratory testing by longitudinally dividing the concrete slices into four layers (Figure 3). The individual layers were numbered 1 to 4, with sample 1 corresponding to the annular cross section’s inner layer and sample 4 to its outer layer (Figure 3b). Additionally, a whole concrete slice with its individual layers unseparated (Figure 3b) constituted sample 5.

For aggregates with a maximum particle diameter of up to 16 mm, the mass of the concrete sample to be tested should be at least 3 kg [38]. Due to the size of the spun concrete samples, this condition was not met. Moreover, during the cutting of concrete slices, it turned out to be impossible to completely separate the individual layers of the material structure (Figure 3a); hence, small fragments of adjacent layers may be present in some parts.

## 3. Test Methods for Determining Hardened Concrete Composition

To test the composition of hardened concrete, an approximate method, based on determining the apparent density of concrete, the HCl-insoluble part content, the limestone aggregate content and the amount of components attached to the binder during its hardening, was used in accordance with instructions [38]. The method described in [38] had been validated by the test results reported in [36,37].

As already mentioned, the adopted method has certain limitations. First, the individual structural layers could not be completely separated during sample cutting, and small fragments of adjacent layers may therefore have been included in individual test sections. Second, it was not possible to obtain test specimens of the required mass. The extent of the deviation in the test results caused by the mixing of layers during cutting was not determined, and the systematic error associated with the method was not quantified. Due to the small number of specimens available for testing, no statistical analysis of the results was performed.

The apparent density (*ρ_c_*) of the concrete, defined as the ratio of the mass of the dry sample to its total volume (including air voids), was determined using the hydrostatic method because of the irregular shape of the samples. The determination was performed according to [43]. The samples were dried at 105 °C until a constant mass was obtained, i.e., until the mass change within 24 h was less than 0.2%. The dried samples were then weighed. In the next step, the samples were saturated by immersion in water at 20 °C until the mass change within 24 h was less than 0.2%, after which they were weighed again. The volume of each sample was determined on the basis of its apparent mass in water and mass in air from the following relationship:

*V* = (*m_a_* − *m_w_*)/*ρ_w_*, (1)

where *m_a_* is the saturated sample’s mass in air, *m_w_* is its apparent mass in water, and *ρ_w_* = 998 kg/m^3^ is the density of water at 20 °C. The apparent density of the dry concrete was calculated as:

*ρ_c_* = *m*_0_/*V*, (2)

where *m*_0_ is the mass of the dry sample. At the same time, the absorption capacity by weight of the particular samples was calculated:

*n_w_* = (*m_a_* − *m*_0_)/*m*_0_. (3)

The actual density (*ρ_cr_*) of the concrete, defined as the ratio of the mass of the dried sample to the volume of the solid part (excluding the volume of voids), was determined using the Le Chatelier flask method [44]. Using the values of *ρ_c_* and *ρ_cr_* the total porosity was calculated from the formula:

*ϕ*_*p*,*tot*_ = 1 − (*ρ_c_*/*ρ_cr_*) = 1 − *S*, (4)

where S stands for the watertightness of the material. When the tightness is 100%, it means the material is pore-free. With decreasing tightness, the probability of occurrence of open pores and higher water absorption of the material increases. Open porosity *ϕ_p_*_,*op*_ was estimated on the basis of the water absorption capacity by weight of the concrete from the relationship:

*ϕ*_*p*,*op*_ = *n_w_* · (*ρ_c_*/*ρ_w_*). (5)

The total aggregate content in concrete is assumed to be equal to the hydrochloric acid (HCl)-insoluble part content. Since dolomite aggregate was used in the production of the ETG-1 pole, the aggregate content analysis was conducted in two stages. In the first stage, the HCl-insoluble aggregate content was determined. In the second stage, the limestone aggregate content was determined, assuming that the aggregate decomposes in the temperature range of 580–1000 °C according to the reaction: CaCO_3_ = CaO + CO_2_. In both stages, the determinations were performed in parallel on two samples, and the final result was the arithmetic mean of the obtained values. Because of the specific nature of spun concrete samples 1–5, the required sample mass of approximately 1 kg could not be maintained. After grinding, sifting through a 1 mm sieve and drying to constant mass at 105 °C, the samples were passed through a 0.2 mm sieve.

In the first stage of the investigation, analytical samples, each weighing approximately 2 g, were analysed using an aqueous solution of hydrochloric acid (HCl) (1:3) to determine the insoluble part content. The sample was placed in a 250 mL beaker, and 100 mL of room-temperature HCl solution was added. The contents of the beaker were stirred, and the insoluble residue was triturated with a glass rod. After 15 min, the supernatant was removed by decantation.

Then 50 mL of HCl solution was added to the precipitate, and the sample was placed in a water bath at 90 °C for 15 min. After this time, the contents of the beaker were washed twice with hot water and decanted. The precipitate remaining in the beaker was poured into 50 mL of 5% Na_2_CO_3_ solution and placed in a water bath at 90 °C for 15 min, then washed twice with hot water and decanted.

The remaining precipitate was poured into 50 mL of water and acidified with HCl solution (1:3) in the presence of methyl orange, adding an excess of 3–4 drops of the acid to the neutralized solution. The contents of the beaker were then filtered. The precipitate from the filter was washed six times with hot water until the chloride reaction ceased (controlled by the reaction of the precipitate with AgNO_3_).

The washed precipitate was transferred to a previously weighed porcelain crucible, and after filter combustion it was calcined at 1000 °C. On this basis, the average mass of HCl-insoluble parts was determined, and then the insoluble part percentage C_ins,%_ was calculated.

It was assumed that the aggregate (sand) content in the concrete in the individual layers is directly equal to the HCl-insoluble part content, i.e., C_agg,s%_ = C_ins,%_. The HCl-insoluble part content in kg/m^3^ was determined according to the formula:

*C*_*agg*,*s*_ = (*C*_*agg*,*s%*_/100) · *ρ_c_*. (6)

In the second stage of the investigation, the weight loss percentage *A*_580+1000_ caused by limestone aggregate decomposition during the calcination of the samples, first at *t*_1_ = 580 °C and then at *t*_2_ = 1000 °C, was determined:

*A*_580+1000_ = (*m*_0_ − *m_cal_*_,580+1000_) · 100*/m*_0_, (7)

where: *m_cal_*_,580+1000_—the weight of the sample calcinated at temperatures *t*_1_ and *t*_2_. The limestone aggregate percentage was calculated from the formula [38]:

*C_agg_*_,*d*%_ = 2.27 · (*A* − 2.5), (8)

and then the limestone aggregate content in kg/m^3^ in the individual layers was calculated from the relation:

*C_agg_*_,*d*_ = (*C_agg_*_,*d*%_/100) · *ρ_c_*. (9)

The total aggregate content in the samples was determined from the formula:

*C_agg_* = *C*_*agg*,*s*_ + *C*_*agg*,*d*_. (10)

The content of compounds attached to cement during the setting and hardening of concrete, *C_att_*_,%_ (including H_2_O and CO_2_ bonding), was determined based on a thermal analysis of a concrete sample containing limestone aggregate. The percentage mass loss *A*_580_ of the sample during heating to a temperature of *t*_1_ = 580 °C was determined, and then the percentage of the attached compounds was calculated according to the formula [38]:

*C*_*att*,%_ = *A*_580_ + 2.5. (11)

The value of *C_att_*_,%_ was converted to kg/m^3^ according to the relation:

*C_att_* = (*C*_*att*,%_/100) · *ρ_c_*. (12)

Cement content *C_cem_* in the individual samples was calculated from the condition:

*C_cem_* = *ρ_c_* − *C_agg_* − *C_att_*. (13)

## 4. Test Results and Discussion

**Figure 4:**
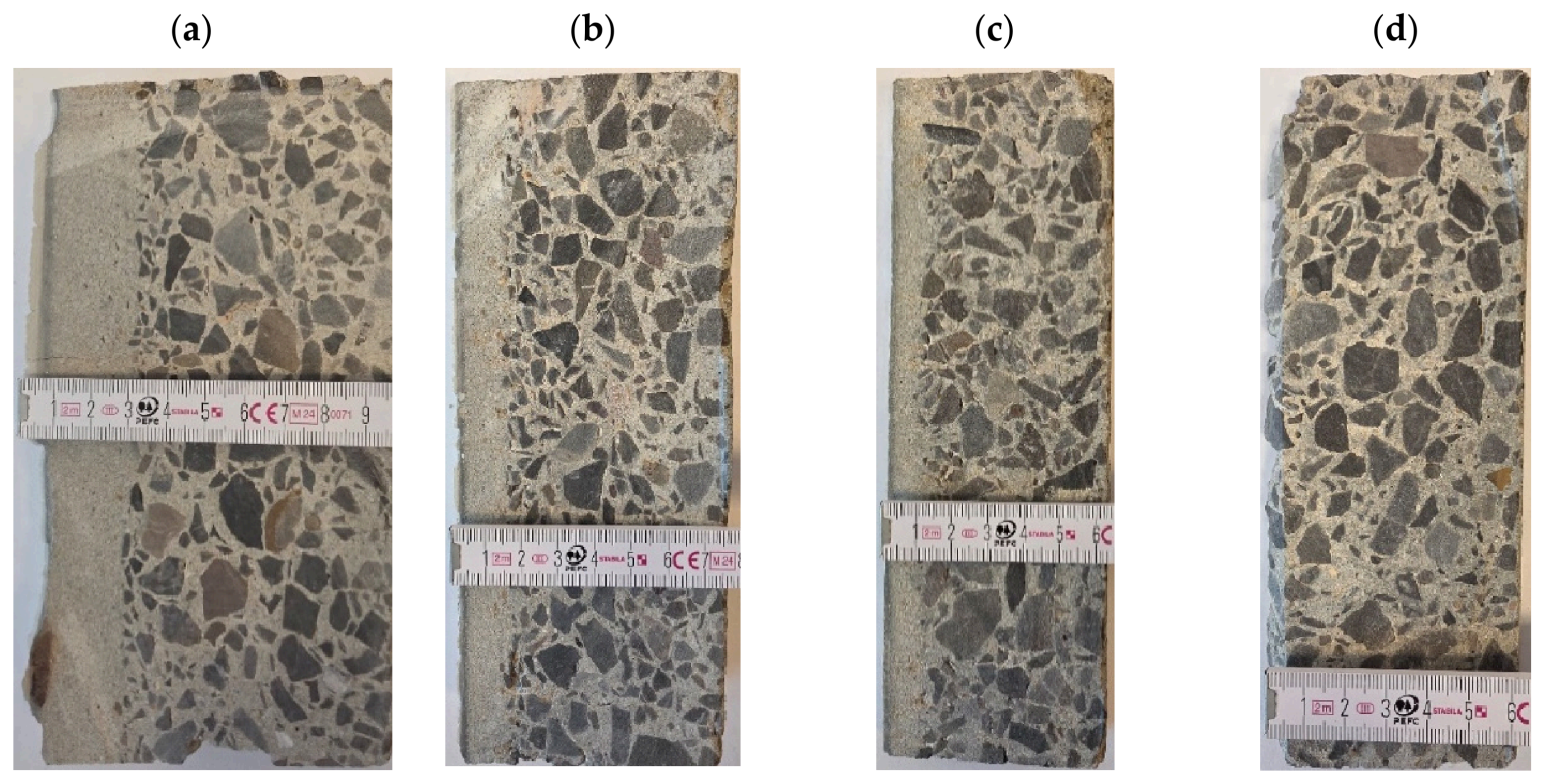
Samples prior to separation into layers: (**a**) sample A, (**b**) sample B, (**c**) sample C and (**d**) sample D.

**Figure 5:**
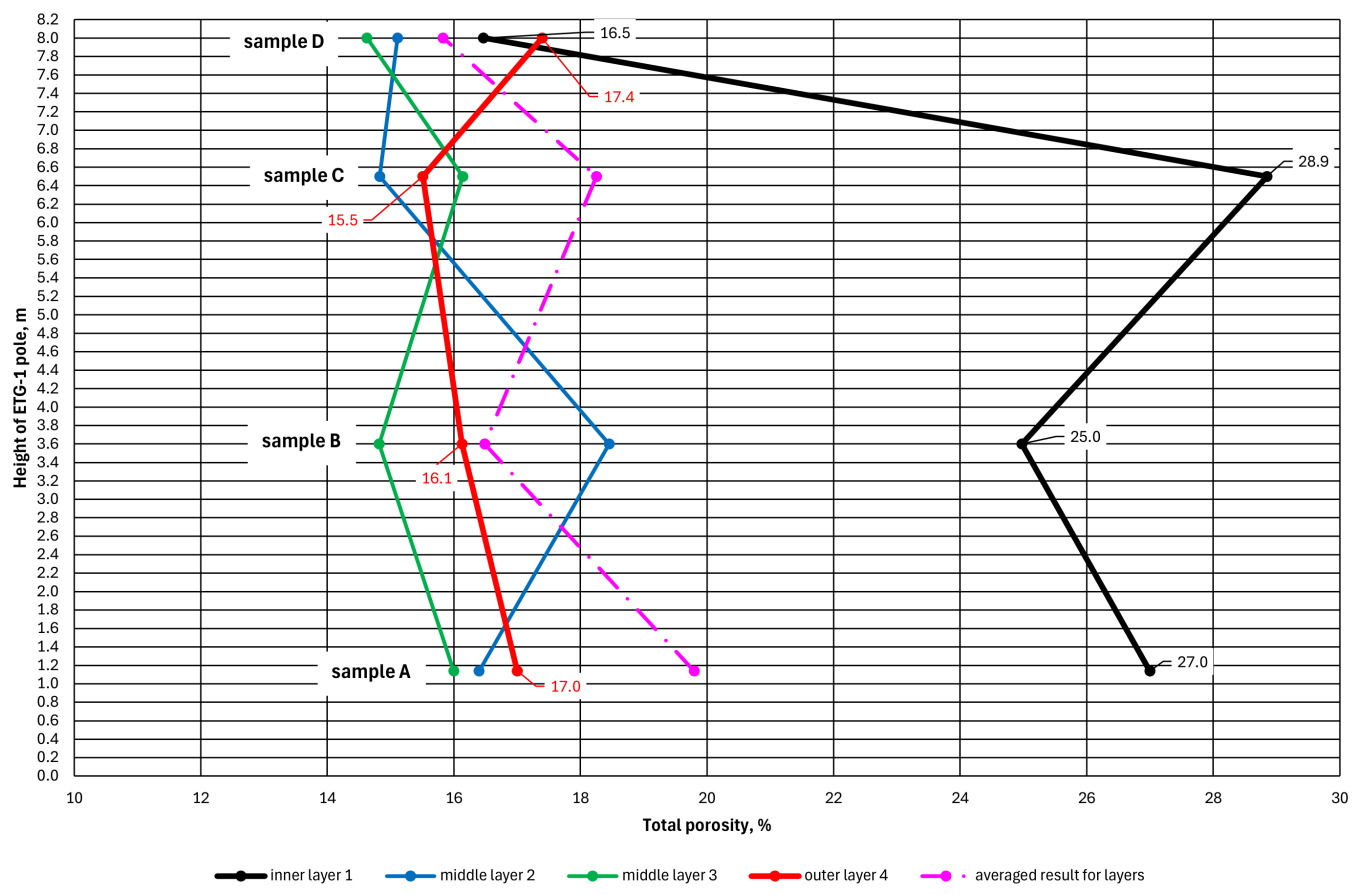
Variation of total porosity *ϕ_p_*_,*tot*_ in individual cross-section layers (1 ÷ 4 and averaged layer 5) along pole height.

**Figure 6:**
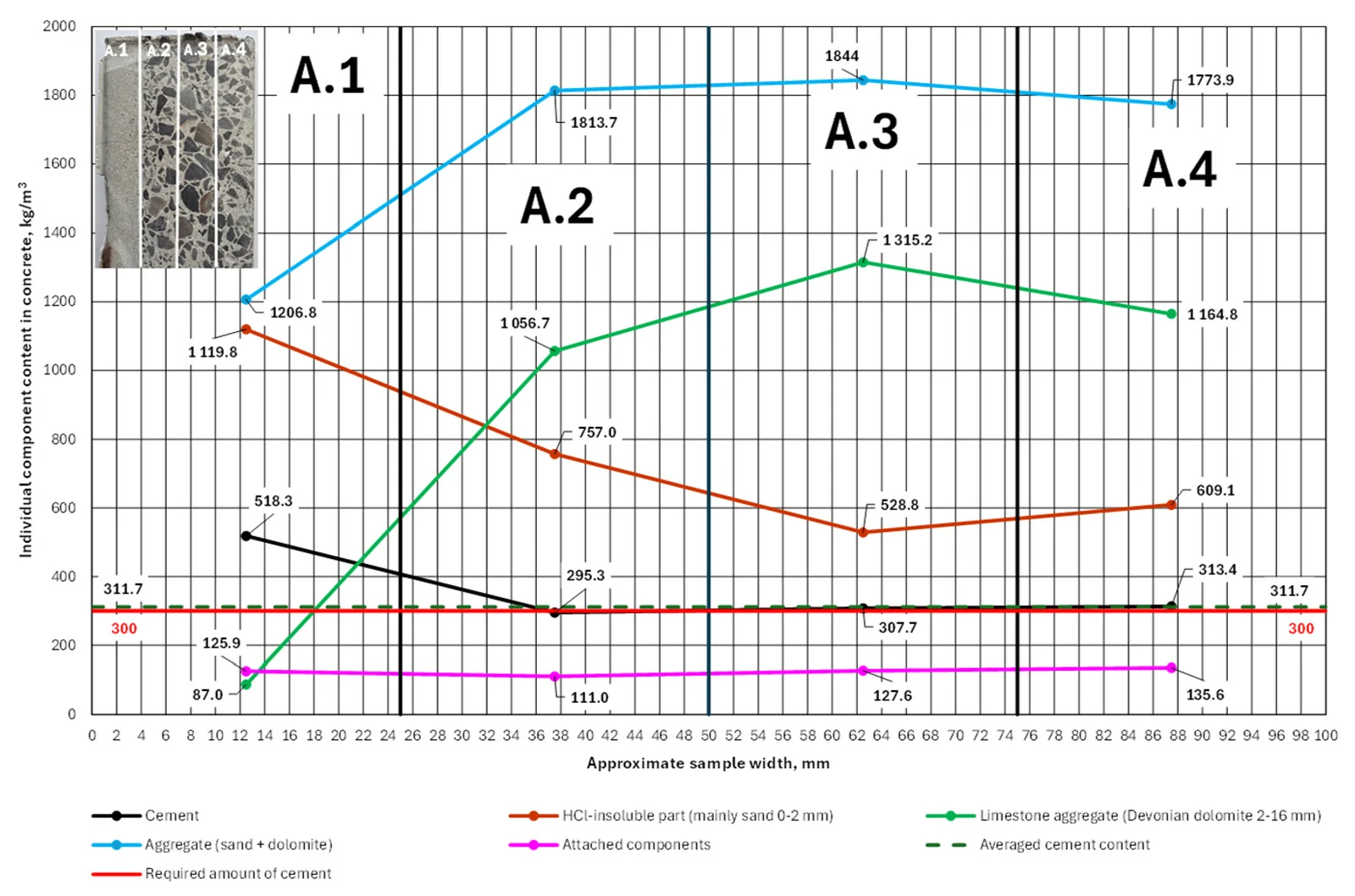
Individual components content in concrete for samples A.1 ÷ A.4 and averaged sample A.5, taken at distance of 7.15 m from top of ETG-1 pole.

**Figure 7:**
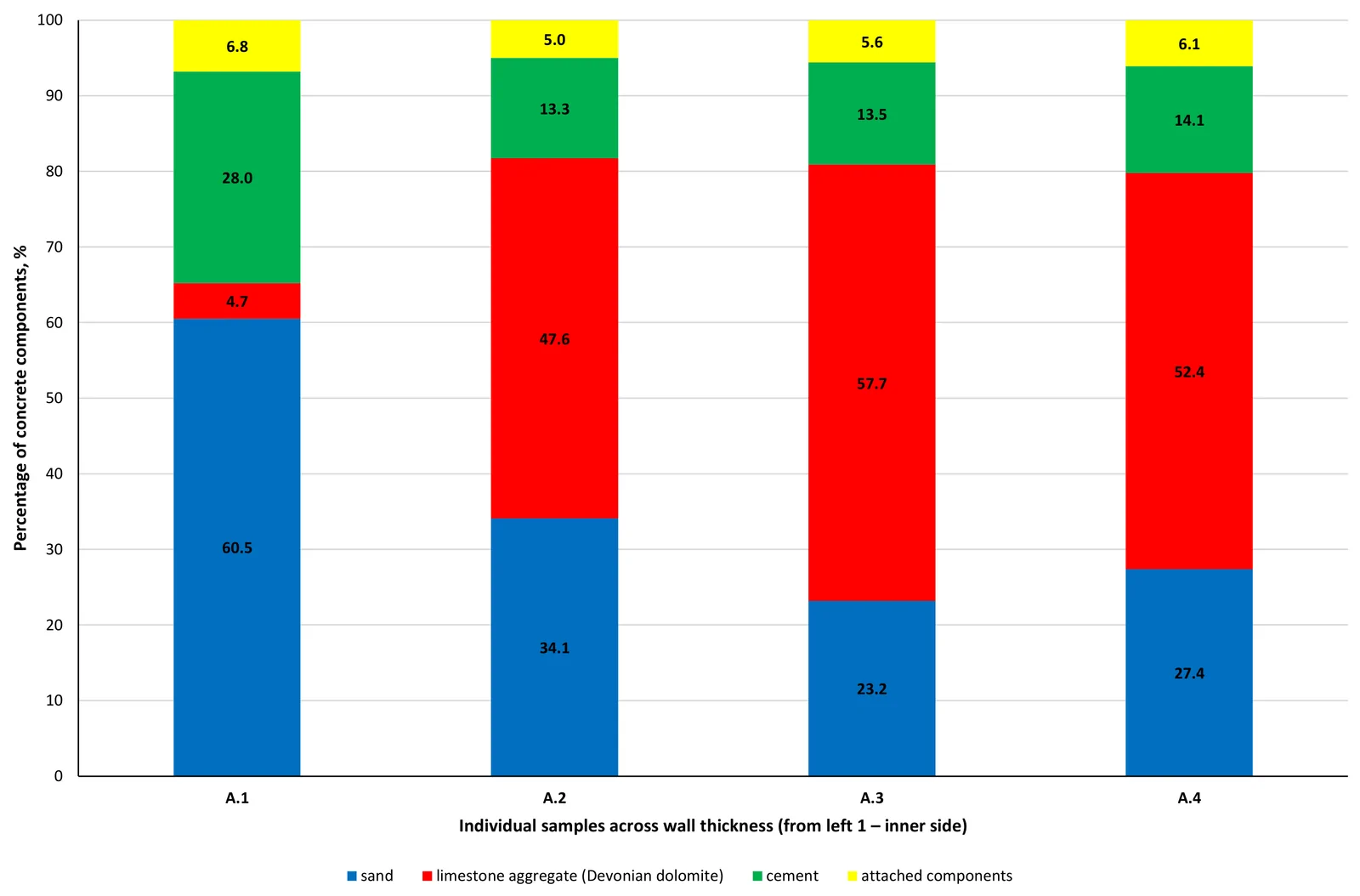
Percentage of individual concrete components for samples A.1 ÷ A.4 taken at distance of 7.15 m from top of ETG-1 pole.

**Figure 8:**
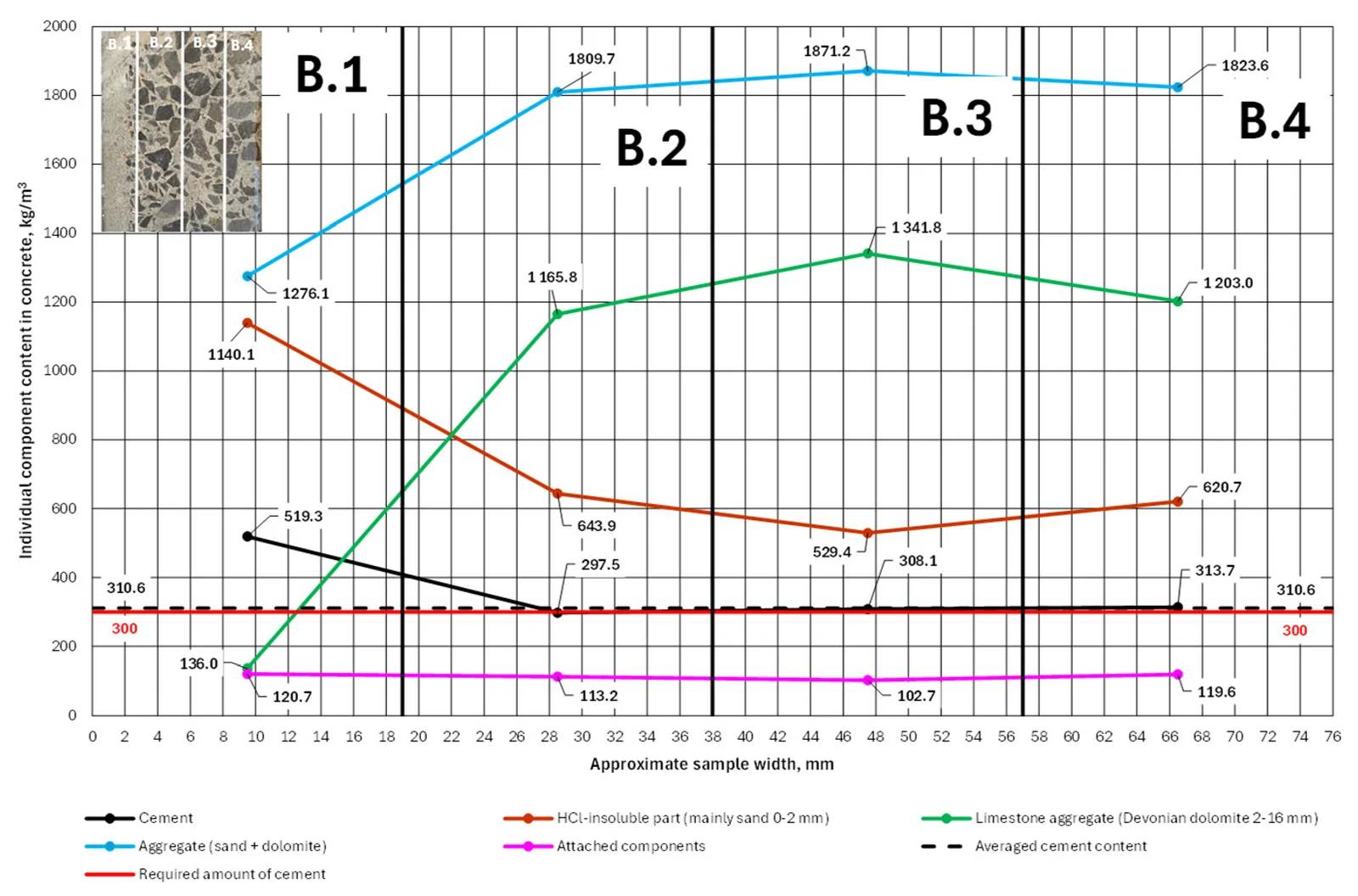
Individual components content in concrete for samples B.1 ÷ B.4 and averaged sample B.5, taken at distance of 4.70 m from top of ETG-1 pole.

**Figure 9:**
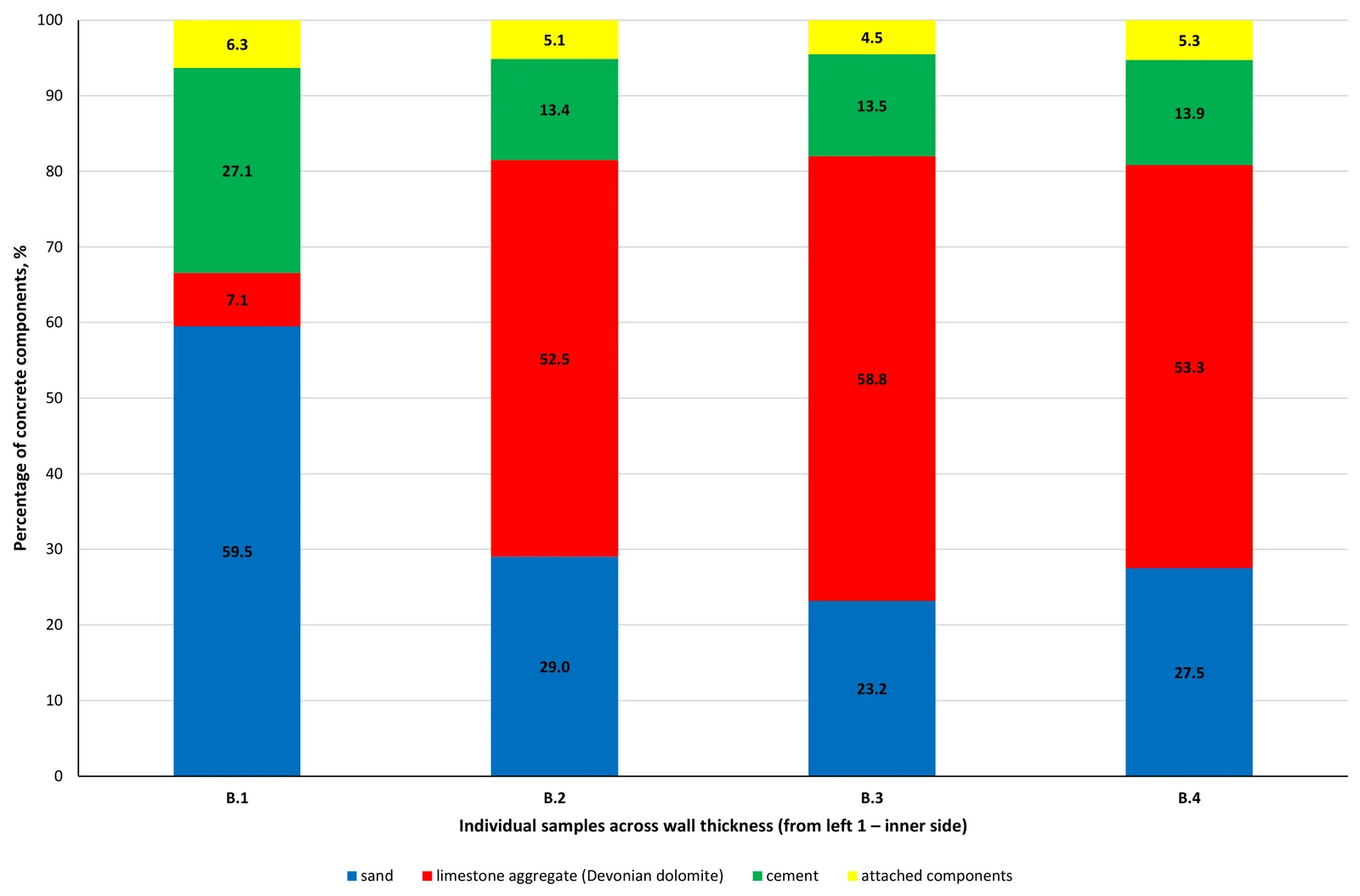
Percentage of individual concrete components for samples B.1 ÷ B.4 taken at distance of 4.70 m from top of ETG-1 pole.

**Figure 10:**
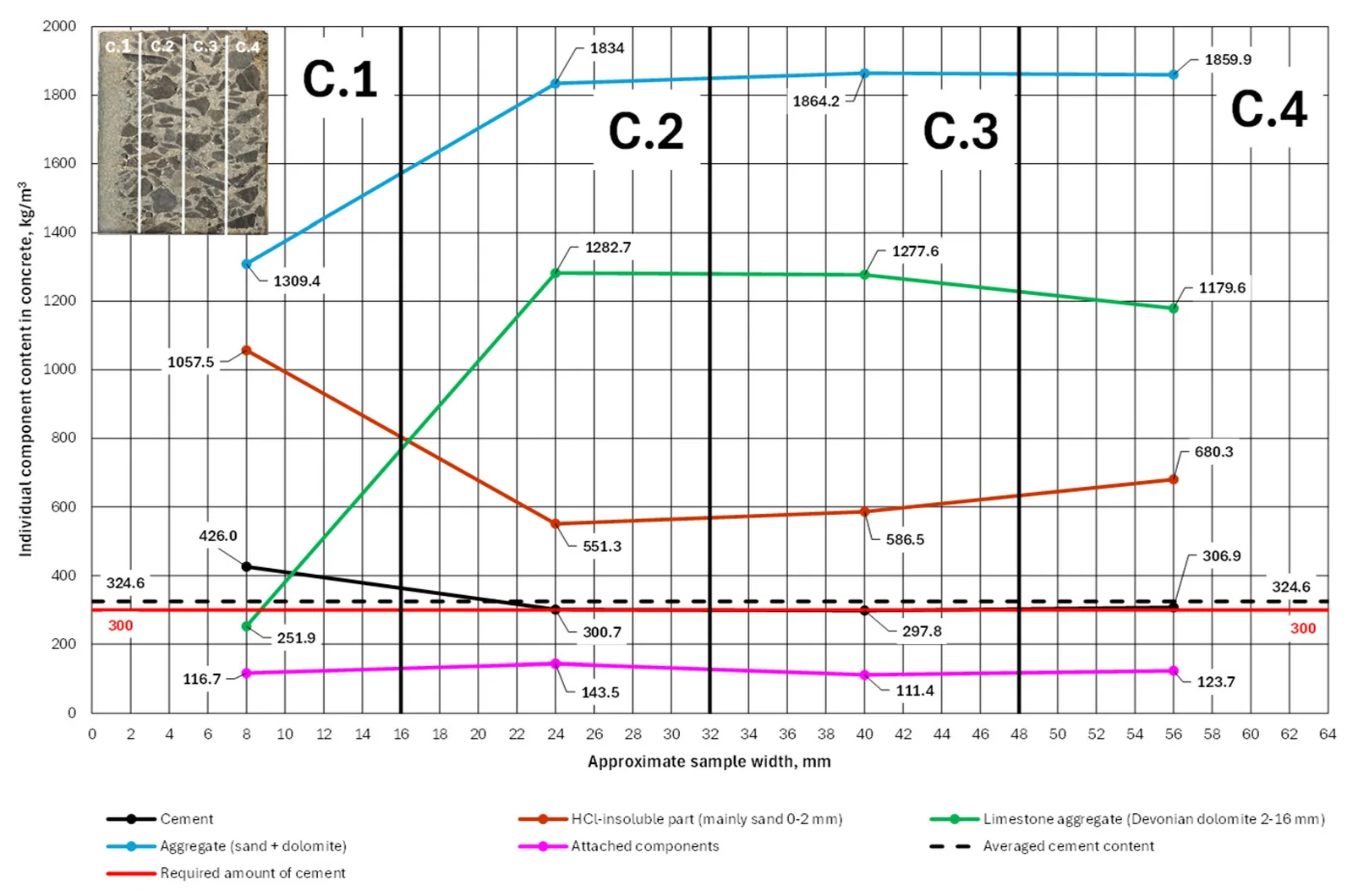
Individual concrete components for samples C.1 ÷ C.4 and averaged sample C.5, taken at distance 1.80 m from top of ETG-1 pole.

**Figure 11:**
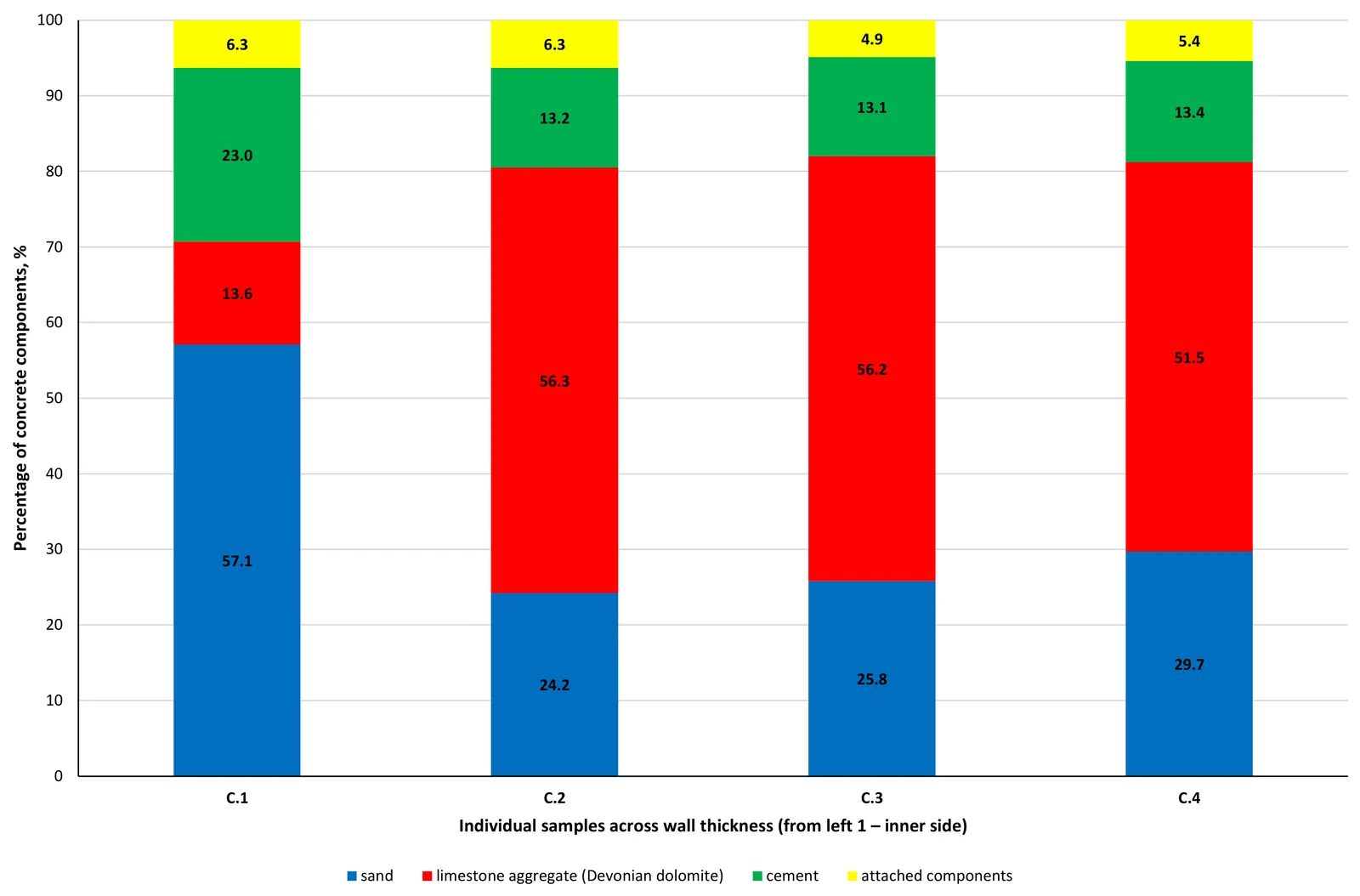
Percentage of individual concrete components for samples C.1 ÷ C.4 taken at distance of 1.80 m from top of ETG-1 pole.

**Figure 12:**
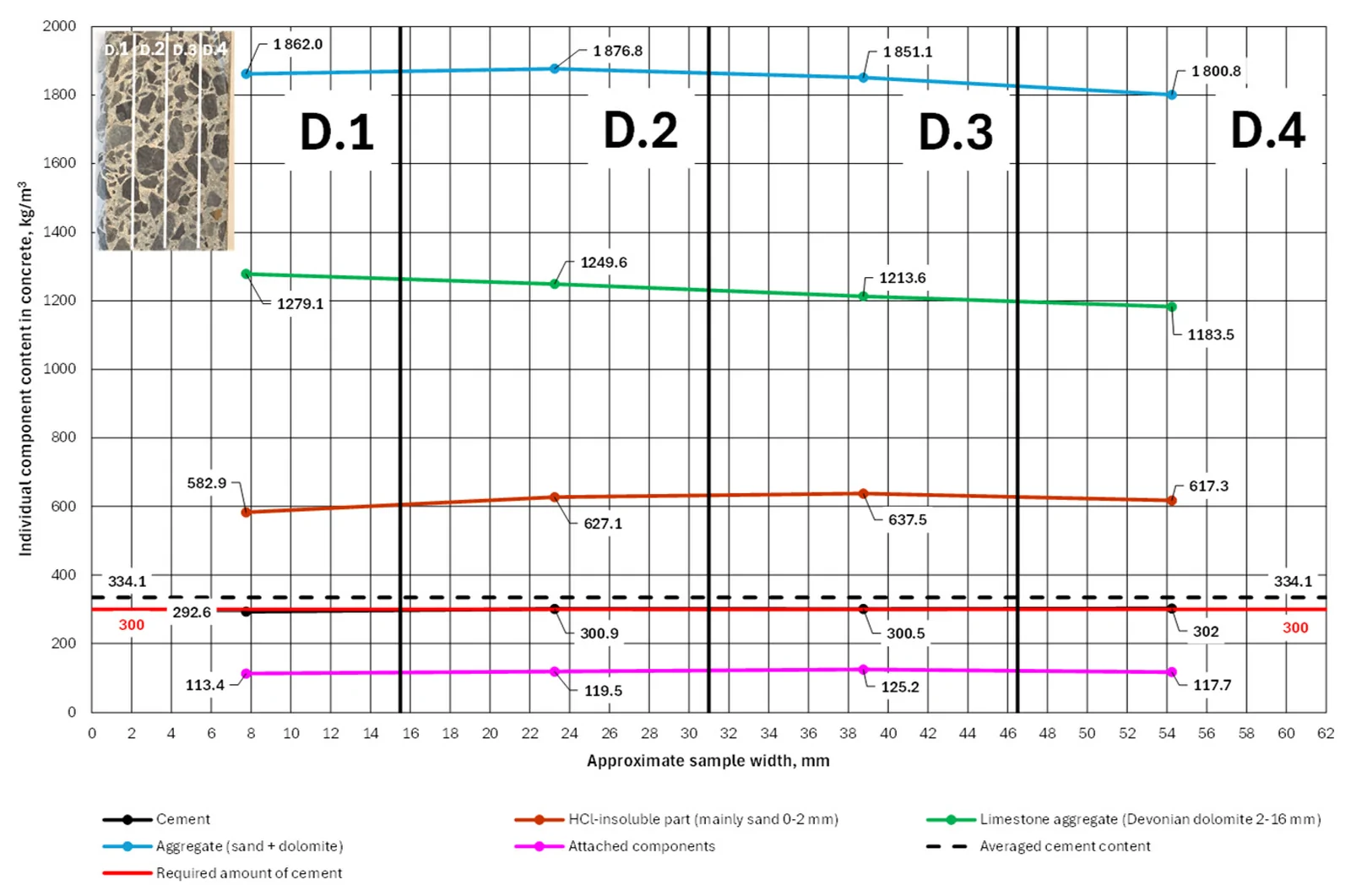
Individual components content in concrete for samples D.1 ÷ D.4 and averaged sample D.5, taken at distance of 0.30 m from top of ETG-1 pole.

**Figure 13:**
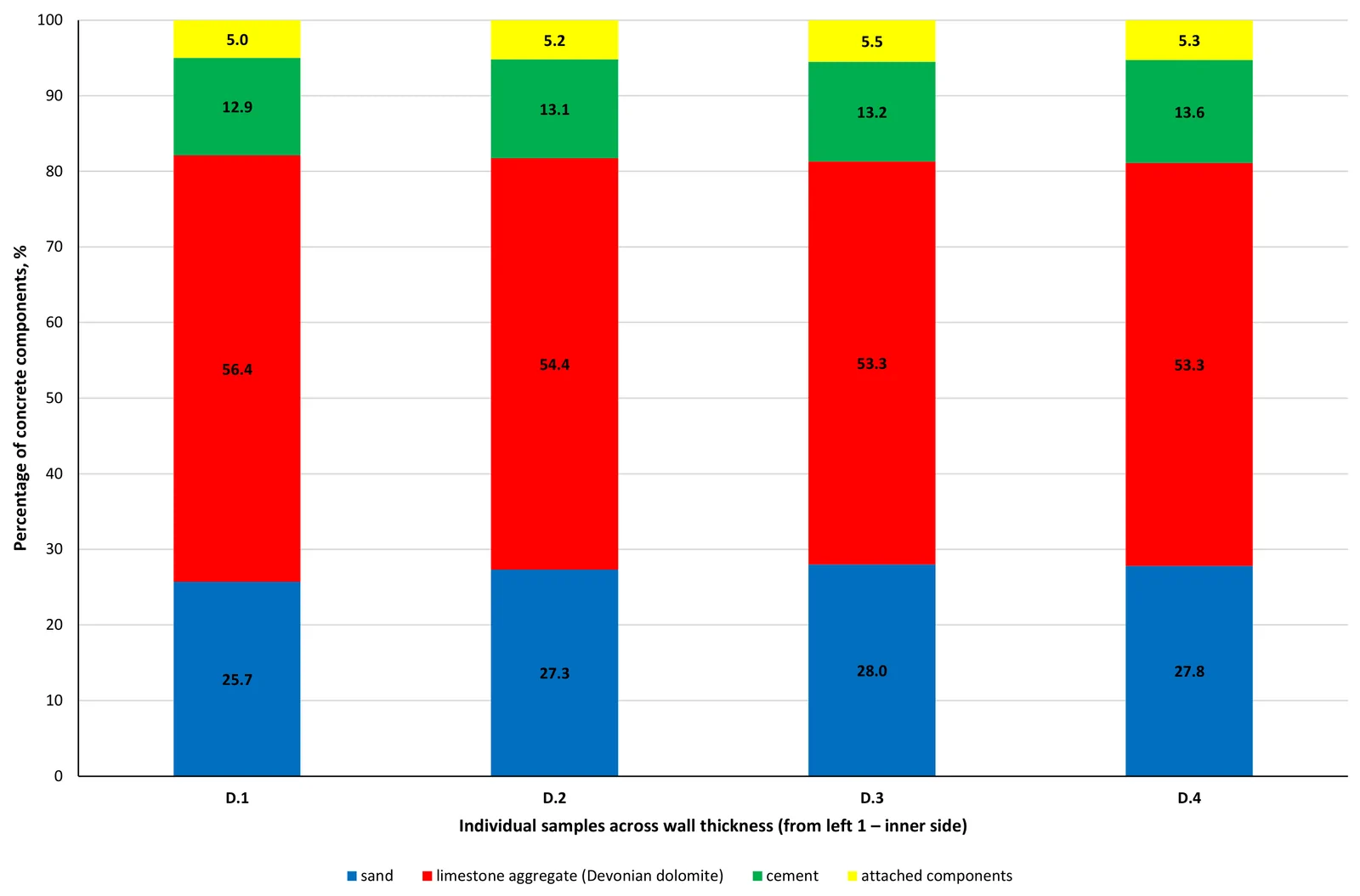
Percentage of individual concrete components for samples D.1 ÷ D.4 taken at distance of 0.30 m from top of ETG-1 pole.

It should be noted that for individual samples A-D the separated layers (1–4) were characterized by different thicknesses, which resulted primarily from the actual thickness of the cross-section wall (Figure 4). Figure 5 shows the variation of total porosity *ϕ_p_*_,*tot*_ along the pole height. The determined parameters of concrete in the particular layers of the samples taken along the height of the ETG-1 pole are presented in Table 3, Table 4 and Table 5 and Figure 6, Figure 7, Figure 8, Figure 9, Figure 10, Figure 11, Figure 12 and Figure 13. In Figure 6, Figure 8, Figure 10 and Figure 12, the individual layers were geometrically separated, and the test results were correlated with the centers of the layers. Since, when cutting the concrete slices, it was not possible to completely separate discrete layers of the material structure, small fragments of adjacent layers could be present in some parts.

It was found (Figure 4) that in the pole’s top part (sample D) the concrete structure was less stratified than towards its base (sample A). In sample A, the thickness of the inner layer, consisting mainly of cement paste and sand concrete, was approximately 30 mm, which corresponds to about 30% of the wall thickness. In sample B, this value was approximately 15 mm (about 20%), while in sample C it was approximately 10 mm (about 16%)—Figure 4. Minor shrinkage cracks were observed in the inner layer of sample A (Figure 4a). In sample D, it was not possible to separate a discrete inner layer.

The inner surfaces of samples A–C, coated with a thin layer of cement laitance, were relatively smooth. The irregularities visible in Figure 4a–c resulted mainly from the cutting process. In sample D, the inner surface was clearly more irregular, which was due to the lack of clear cross-sectional layering and the presence of coarse aggregate in this zone (Figure 4d). The percentage of sand in the total aggregate mass exceeded 37% (Table 2), and so it was greater than 25%, which, according to the research results presented in [26,27,28], favours concrete structure segregation.

In the pole’s top part, the cross-section wall thickness met the design assumptions, while towards its base it increased beyond the design values. Technologically, it is very difficult to obtain the required wall thickness of a spun concrete pole [45]. Reducing the wall thickness in the pole’s top part to below 60 mm can be hazardous due to the high probability of material deficiencies, such as superficial and longitudinal cracks [22], resulting from exceeding stresses in the concrete in the initial stage (when the pole is being prestressed) or from a failure to meet the requirements for the minimum reinforcement cover from the side of the interior of the pole’s tubular cross section. The minimum cross-section wall thickness in the pole’s middle part and at its base should amount to 75 mm [45]. Increased wall thickness in the pole’s base part, exceeding the design requirements, is acceptable and results from the use of a semiliquid concrete mix during production. This type of mix is introduced into the mould using a pneumatic pump [42].

The inner concrete layer across the wall thickness is characterized by significantly higher porosity (Figure 5) and higher water absorption (Table 3, Table 4 and Table 5) along almost the entire height of the pole in comparison with the other layers. These results corroborate the observations presented earlier in the literature [23,24,27,28,29,32,34,35]. Sample D (Table 6), taken from the top part of the pole, had a clearly different character. In this case, both porosity and water absorption were relatively uniform across the entire wall thickness of the cross section (Figure 5). This confirms that the material structure in this part of the pole is more homogeneous (Figure 4d).

The lack of clear concrete mix segregation in the pole’s top part can be explained by the proper matching of the centrifugation parameters (time and speed) to the smallest radius of the tubular mould and a significantly smaller amount of fine ingredients, including sand, which were naturally moved from the top towards the base due to the use of a semiliquid mixture.

The graphs in Figure 6, Figure 7, Figure 8, Figure 9, Figure 10 and Figure 11 show that coarse aggregate (2–16 mm) predominated in the outer layers, while the inner layers contained a much smaller proportion (in places approaching zero) of this aggregate. The opposite trend was observed for sand (0–2 mm). In places where the coarse aggregate content was the lowest, the voids were filled with cement mortar (cement + sand). It is also interesting that the total aggregate content as a function of wall thickness remained practically constant in all the layers (Figure 6, Figure 7, Figure 8, Figure 9, Figure 10 and Figure 11), except for the inner layer. However, this phenomenon did not apply to sample D taken from the top of the pole (Figure 12 and Figure 13).

The results for aggregate distribution as a function of cross-section wall thickness and macroscopic observations confirm the segregation of coarse particles towards the outer zone of the active cross section and are consistent with the results presented in the literature [31]. The aggregate content determined during the laboratory tests (samples A.5, B.5, C.5 and D.5—Table 3, Table 4, Table 5 and Table 6) was close to the amount of aggregate declared in the concrete mix design (Table 2) provided by the manufacturer of the spun concrete poles.

**Figure 14:**
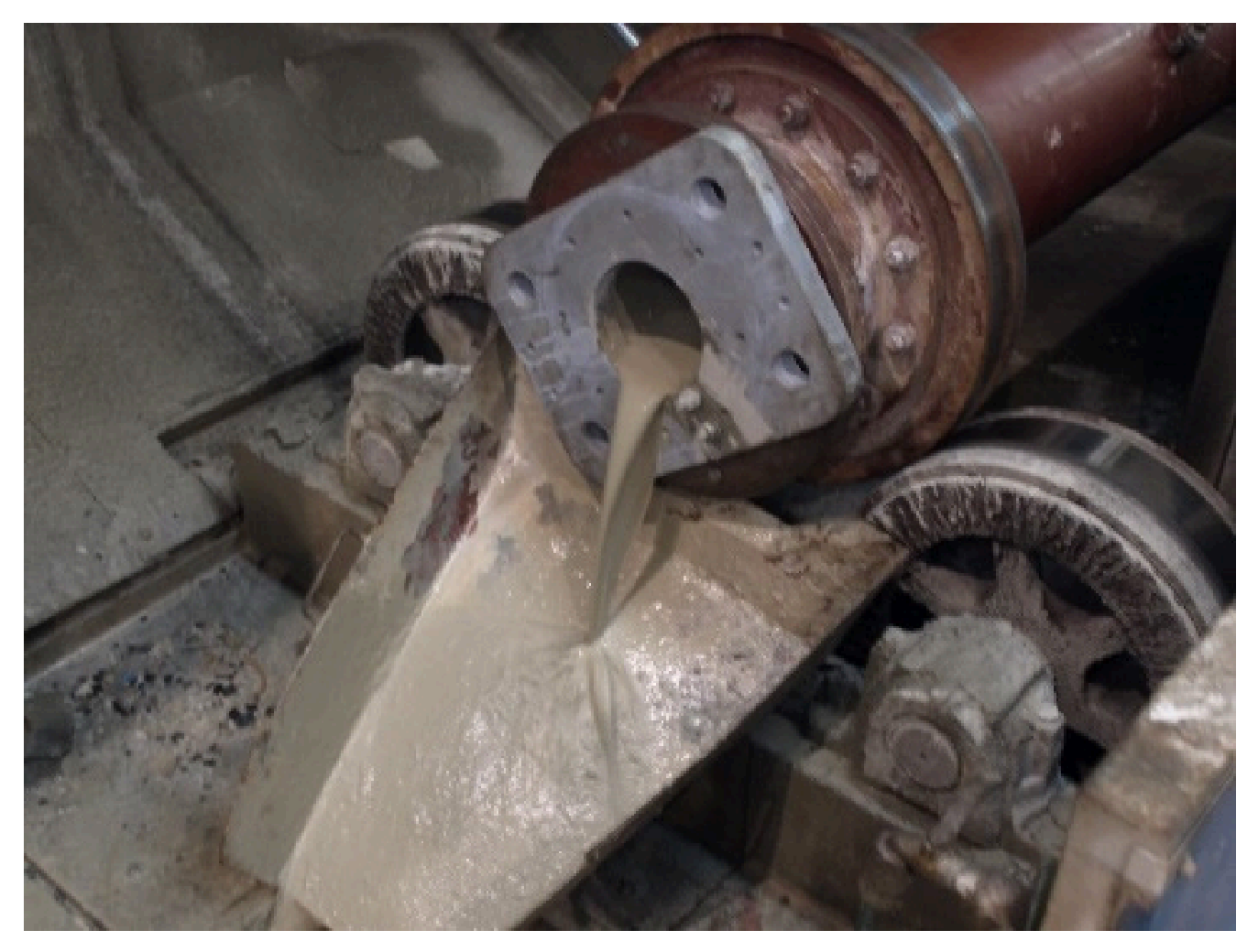
Discharge of bleed water from mould after concrete mix centrifugation.

**Figure 15:**
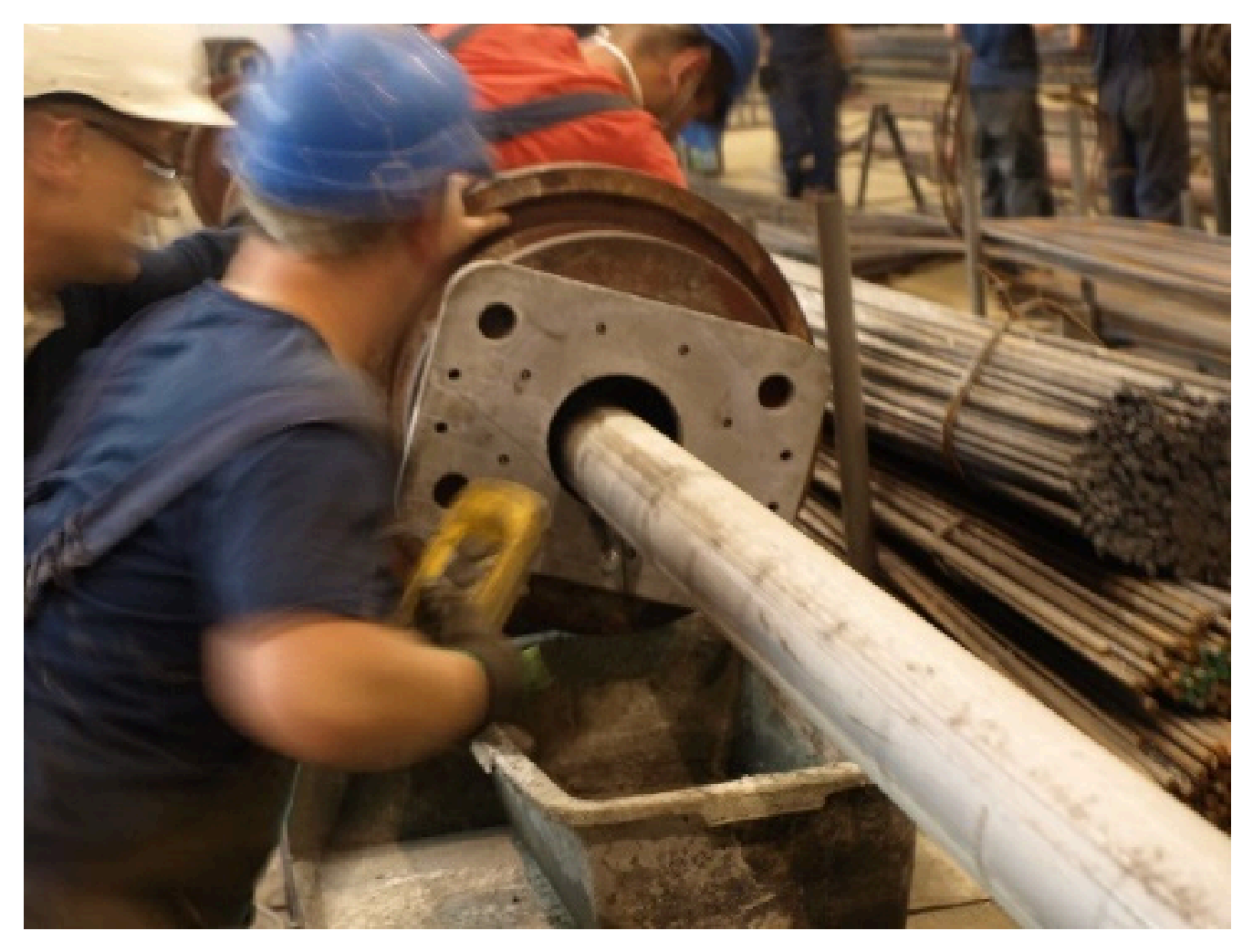
Concrete mix pumping into mould.

Cement content distribution was found to be inhomogeneous in all the analysed samples. The inner layers consistently showed the highest cement content, while the outer layers were characterized by lower values (Figure 6, Figure 7, Figure 8, Figure 9, Figure 10 and Figure 11 and Table 3, Table 4, Table 5 and Table 6). These results are consistent with the conclusions presented by Marquardt [23]. The cement content in the individual layers, determined using the laboratory method (Figure 6, Figure 7, Figure 8, Figure 9, Figure 10 and Figure 11 and Table 3, Table 4, Table 5 and Table 6), was lower than the value of 400 kg/m^3^ declared by the manufacturer of the spun concrete poles in the concrete mix design (Table 2). The obtained results confirm the observations presented in [35]. The noted decrease in cement content can be explained by the discharge of some of the bleed water from the mould after the centrifugation process (Figure 14). Bleed water contains the finest mix ingredients, including cement and evaporable water necessary to obtain the semiliquid consistency necessary in order to pump the mix (Figure 15).

**Figure 16:**
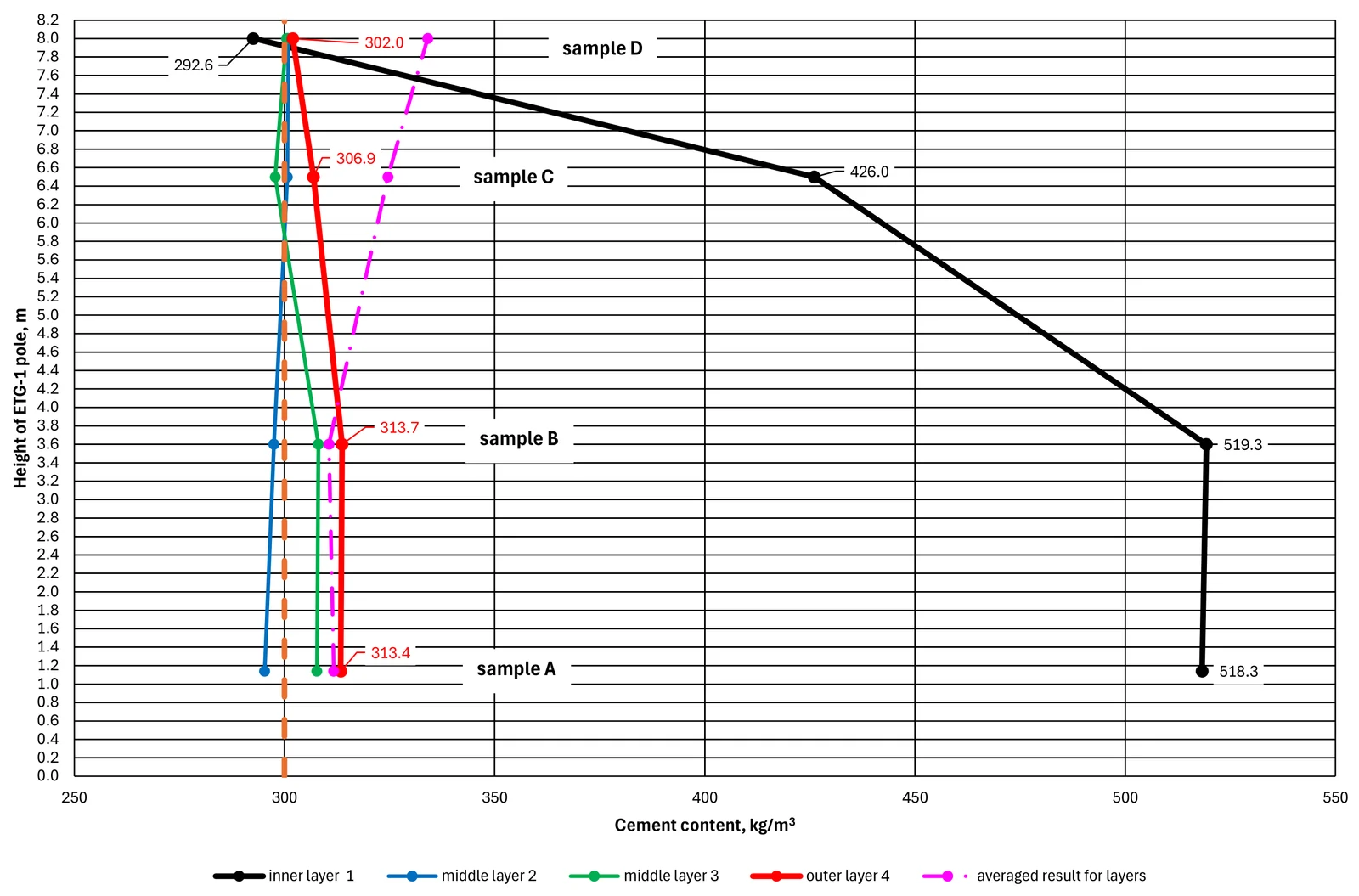
Variation of cement content in individual cross-section layers (1 ÷ 4 and averaged layer 5) along pole height. Orange dashed line represent the code-required amount of cement.

The variation of cement content along the height of the analysed ETG-1 pole is shown in Figure 16. The cement content in the inner layer gradually decreased along the pole height, from approximately 518 kg/m^3^ in the lower and almost central part of the pole (samples A.1 and B.1—Table 3 and Table 4), through 426 kg/m^3^ (sample C.1—Table 5), to approximately 300 kg/m^3^ in the top part of the pole (sample D.1—Table 6). The inner layer clearly differed from the other layers across wall thickness. In the outer layer, the cement content at the height of the pole was slightly over 300 kg/m^3^.

The decrease in cement content observed in sample D.1, both in the outer and inner layers, can be explained by the probable movement of the semiliquid mix from the top towards the base of the pole during the filling and spinning of the mould.

Considering the durability of spun concrete power poles exposed to direct weather conditions, the minimum cement content in the outer layer should not be less than 300 kg/m^3^ [2]. It should be noted here that the standard requirements [2] regarding minimum cement content depending on the environmental exposure class were specified for conventional concretes compacted by vibration. Currently, there are no clear guidelines regarding concretes compacted by other methods, including centrifugal spinning. In the analysed outer layer, as in the other layers, the required minimum cement content was maintained (Figure 16). The obtained results corroborate the long failure-free operation of the spun concrete poles used in Poland for over 30 years [12].

The test results confirm that the centrifugal spinning of concrete mix leads to a significantly uneven distribution of concrete components across the wall thickness of the elements. This observation is consistent with the previous studies indicating that aggregate and cement mortar segregate under the action of centrifugal forces. The present study, however, provides a quantitative assessment of this phenomenon in relation to cement content and material durability parameters.

The key conclusion emerging from this study is that despite the nominal mix design cement content of 400 kg/m^3^, the actual cement distribution across wall thickness is not uniform. The cross section’s inner layers are characterized by increased cement content, while its outer layers exhibit a relatively lower cement amount. At the same time, the outer zones contain a higher proportion of coarse aggregate. This confirms that centrifugal spinning results in a material gradient, and not in a homogeneous material structure as is often assumed in the design process. The observed decrease in the actual amount of cement relative to the nominal amount should prompt the manufacturer to consider technological and material changes in the spinning process, preceded by appropriate testing.

From the durability perspective, this inhomogeneity may be of significant importance. The durability requirements in the current codes are defined in terms of, among other things, the minimum cement content and the *w*/*c* ratio, assuming uniform material properties. The results obtained in this study indicate that such assumptions may not be fully representative of spun-cast concrete elements. In particular, the outer layers, which are most exposed to environmental factors, may locally contain less cement than required by the design criteria, even if the average concrete composition meets the standards. However, this topic should be investigated more comprehensively, as studies of ordinary concrete [46,47] have shown that, at a constant *w*/*c* ratio, reducing the cement content by as much as 22% did not adversely affect most concrete properties and, in some cases, even resulted in improvements. These findings are consistent with those reported by other researchers [46,47,48,49,50,51,52,53], who investigated the influence of various factors on the durability of concrete. Previous studies [46,52] have shown that simply increasing the cement content is not an effective approach to improving the durability of a structural element. Instead, greater emphasis should be placed on the *w*/*c* ratio, as it is directly related to concrete porosity, strength, and durability.

When designing a durable pole structure, it is essential to consider the system as a whole, including the type of cement, water-to-cement ratio, cement content, mineral admixtures, aggregate type and properties, air entrainment, curing, cover, cracking, and exposure class [46,47,48,49,50,51,52,53]. Cement content affects durability both directly and indirectly; however, the resulting durability depends on the interaction between the concrete mix and the service conditions. The cement content and type, as well as the overall mix design, should be selected so that, while maintaining the required *w*/*c* ratio, ensuring proper curing, and providing adequate cover, the concrete achieves the required impermeability, strength, and resistance to specific environmental exposures [46,48].

Therefore, the relationship between cement content and the durability of reinforced concrete is significant but not linear. Simply increasing the cement content per 1 m^3^ of concrete does not guarantee increased durability, and excessive cement content may even contribute to certain types of deterioration. Conversely, insufficient cement content may make it difficult to achieve the required strength and watertightness. Too little cement may result in increased porosity and permeability, insufficient paste to adequately coat and compact around the aggregate particles, and reduced resistance to chloride penetration and carbonation. In well-designed concrete, the cement content should be sufficient to achieve the required microstructure, strength, and durability without being unnecessarily increased. Excessive cement content, as observed in the inner layers of the cross-section, results in a higher volume of cement paste and may consequently lead to greater drying and autogenous shrinkage, greater heat of hydration, and higher internal stresses. These effects may contribute to cracking of the concrete (Figure 1).

This finding is crucial for exposure classes such as XC4 and XD1, where resistance to carbonation and chloride ingress depends largely on the quality and composition of the concrete cover. Reduced cement content in the outer layer can lead to higher permeability and lower durability, potentially accelerating degradation processes. At the same time, the inner layers, despite the higher cement paste content, exhibit higher porosity and water absorption, which can also negatively impact the long-term performance of the element.

The test results have also shown that the degree of segregation varies along the height of the element. In the upper part of the pole, the material structure was more homogeneous, and the differences between layers were less pronounced. Whereas in the lower parts, clear concrete stratification was observed, with a well-developed inner layer composed mainly of cement paste and fine particles. These results suggest that technological parameters, such as rotational speed, centrifugation time, and element geometry, have a significant impact on the final material structure.

From a practical perspective, the obtained results indicate that the durability assessment of spun concrete elements should not be based solely on the nominal parameters of the concrete mix. It seems necessary to take into account the actual distribution of components across wall thickness, particularly with regard to the properties of the reinforcement cover zone. This may require a revision of the current design assumptions or the introduction of additional safety factors when assessing the durability of such elements. It should also be noted that, in addition to the quality control of the composite itself, stresses and associated microcracks arising during the transport of precast poles from the factory to the installation site [54,55,56] and during the service life of the elements [22,40] can have a significant impact on their durability.

Finally, it should be noted that the method used to determine the composition of hardened concrete has certain limitations, primarily connected with the small dimensions of the samples and the difficulty of separating perfectly homogeneous material layers. Nevertheless, the consistency of the results obtained for numerous samples and their agreement with the findings reported in the literature confirm the validity of the formulated conclusions.

## 5. Conclusions

This study investigated the distribution of cement content and selected durability-related parameters across the wall thickness of spun-cast concrete poles. Given the case-specific nature of the study, which did not include statistical analysis of the results or an assessment of the effects of various factors on the long-term durability of the poles, the findings should be regarded as those of a case study. Based on the experimental results, the following conclusions can be drawn:

1. The centrifugal spinning of concrete mix causes an uneven distribution of concrete components across wall thickness, which results in the formation of a layered structure with clear material gradients.
2. Despite the nominal mix design cement content (400 kg/m^3^), the actual cement content varies significantly in the cross section. The inner layers are rich in cement paste, while coarse aggregate predominates in the outer layers, and the latter may contain locally reduced amounts of cement.
3. The cement content in the outer layer of the analysed pole cross-section was found to exceed the standard requirement of 300 kg/m^3^.
4. The outer zone of the cross-section, which is critical to durability, may not exhibit the degree of material homogeneity assumed in standard design approaches, even when the overall concrete mix parameters meet the standard requirements. Further laboratory testing is therefore needed to investigate the effects of different centrifugation parameters and mix proportions.
5. The inner layers show increased porosity and water absorption, indicating a varied microstructure which may also affect long-term durability.
6. The degree of material segregation varies along the height of the element. More pronounced segregation of the constituents was observed in the lower sections of the pole, whereas the upper sections exhibited a more homogeneous structure. The results indicate that the adopted production technology for spun poles does not ensure a uniform cross-section throughout the height of the pole.
7. The results indicate that assessing the durability of spun concrete elements on the basis of solely nominal mix parameters (e.g., cement content, *w*/*c* ratio) may prove insufficient. The actual distribution of components throughout the wall thickness should be taken into account.
8. The presented experimental methodology, although subject to limitations relating to sample preparation and layer separation, has proved effective in identifying trends in the distribution of concrete components, and it can be used in further research.
9. In future studies, more comprehensive laboratory testing should be conducted on cast specimens of spun concrete, including assessments of shrinkage, freeze-thaw resistance, and the effects of spinning-process parameters, to obtain more reliable results. It will also be important to correlate these findings with the production technology used in precast concrete plants.

Summing up, the study demonstrates that when designing spun-cast concrete elements with regard to durability, they cannot be considered as materially homogeneous systems. The identified gradients in cement content and porosity must be taken into account when assessing the long-term performance of such elements.

## Figures and Tables

**Table 1:** Minimum cover c_min_ based on environmental conditions for non-prestressed and prestressed steel in spun concrete poles and towers.

Exposure Class	Minimum Cover Thickness c_min_ [mm] with Regard to Environmental Conditions			
	Prestressed Reinforcement		Conventional Reinforcement	
	≤C40/50	≥C40/50	≤C40/50	≥C40/50
acc. to EN 1992-1-1:2004+AC:2008 [1] for design life span of 50 years				
XC4	35	30	25	20
XD1	40	35	30	25
acc. to EN 13369:2023 [21] for design life span of 50 years				
XC4	35	30 (25) * (20) **	25	20 (15) * (10) **
XD1	40	35 (30) * (25) **	30	25 (20) * (15) **
*—concrete strength class ≥ C40/50 and water absorption of concrete ≤ 5.5% **—concrete strength class ≥ C50/60 and water absorption of concrete ≤ 4.5%				
the author proposes for prestressing steel and ordinary steel acc. to EN 12843:2008 [4] for the design life span of 30 years				
XC4	30 (25) ***	25 (20) ***	20 (15) ***	15 (15) ***
XD1	35 (30) ***	30 (25) ***	25 (20) ***	20 (15) ***
***—concrete strength class ≥ C50/60 and water absorption of concrete ≤ 3.5%				

**Table 2:** Design of concrete mix used in pole ETG-1 production.

Constituent	Amount of Concrete Mix Constituents [kg/m^3^] in Natural Condition
sand 0 ÷ 2 mm	720
crushed stone (Devonian dolomite) 2 ÷ 8 mm	500
crushed stone (Devonian dolomite) 8 ÷ 16 mm	710
cement CEM I 42.5R	400
superplasticizer	2.0
water	152
*w*/*c* ratio	0.38

**Table 3:** Test and calculation results for samples A.1 ÷ A.4 and averaged sample A.5, taken at a distance of 7.15 m from the top of ETG-1 pole.

Parameter		Samples				
		A.1	A.2	A.3	A.4	A.5
Apparent density *ρ_c_* [kg/m^3^]		1851	2220	2279	2223	2150
Specific gravity *ρ_cr_* [kg/m^3^]		2535	2655	2714	2679	2681
Absorption capacity by weight *n_w_* [%]		8.1	4.2	3.7	4.1	4.9
Open porosity *ϕ_p_*_,*op*_ [%]		15.0	9.4	8.5	9.0	10.5
Total porosity *ϕ_p_*_,*tot*_ [%]		27.0	16.4	16.0	17.0	19.8
Tightness *S* [%]		73.0	83.6	84.0	83.0	80.2
Sand (HCl-insoluble part) content	*C_agg_*_,*s*%_ [%]	60.5	34.1	23.2	27.4	33.5
	*C_agg_*_,*s*_ [kg/m^3^]	1119.8	757.0	528.8	609.1	720.1
Limestone aggregate content	*C_agg_*_,*d*%_ [%]	4.7	47.6	57.7	52.4	46.6
	*C_agg_*_,*d*_ kg/m^3^]	87.0	1056.7	1315.2	1164.8	1001.7
Aggregate content	*C_agg_*_,%_ [%]	65.2	81.7	80.9	79.8	80.1
	*C_agg_* [kg/m^3^]	1206.8	1813.7	1844.0	1773.9	1721.8
Attached components content	*C_att_*_,%_ [%]	6.8	5.0	5.6	6.1	5.4
	*C_att_* [kg/m^3^]	125.9	111.0	127.6	135.6	116.1
Cement content	*C_cem_*_,%_ [%]	28.0	13.3	13.5	14.1	14.5
	*C_cem_* [kg/m^3^]	518.3	295.3	307.7	313.4	311.7

**Table 4:** Test and calculation results for samples B.1 ÷ B.4 and averaged sample B.5, taken at distance of 4.70 m from top of ETG-1 pole.

Parameter		Samples				
		B.1	B.2	B.3	B.4	B.5
Apparent density *ρ_c_* [kg/m^3^]		1916	2220	2282	2257	2251
Specific gravity *ρ_cr_* [kg/m^3^]		2554	2723	2679	2691	2695
Absorption capacity by weight *n_w_* [%]		7.2	4.2	3.7	3.8	3.9
Open porosity *ϕ_p_*_,*op*_ [%]		13.9	9.4	8.5	8.6	8.8
Total porosity *ϕ_p_*_,*tot*_ [%]		25.0	18.5	14.8	16.1	16.5
Tightness *S* [%]		75.0	81.5	85.2	83.9	83.5
Sand (HCl-insoluble part) content	*C_agg_*_,*s*%_ [%]	59.5	29.0	23.2	27.5	28.1
	*C_agg_*_,*s*_ [kg/m^3^]	1140.1	643.9	529.4	620.7	632.4
Limestone aggregate content	*C_agg_*_,*d*%_ [%]	7.1	52.5	58.8	53.3	53.0
	*C_agg_*_,*d*_ kg/m^3^]	136.0	1165.8	1341.8	1203.0	1192.8
Aggregate content	*C_agg_*_,%_ [%]	66.6	81.5	82.0	80.8	81.1
	*C_agg_* [kg/m^3^]	1276.1	1809.7	1871.2	1823.6	1825.3
Attached components content	*C_att_*_,%_ [%]	6.3	5.1	4.5	5.3	5.1
	*C_att_* [kg/m^3^]	120.7	113.2	102.7	119.6	114.8
Cement content	*C_cem_*_,%_ [%]	27.1	13.4	13.5	13.9	13.8
	*C_cem_* [kg/m^3^]	519.3	297.5	308.1	313.7	310.6

**Table 5:** Test and calculation results for samples C.1 ÷ C.4 and averaged sample C.5, taken at distance of 1.80 m from top of ETG-1 pole.

Parameter		Samples				
		C.1	C.2	C.3	C.4	C.5
Apparent density *ρ_c_* [kg/m^3^]		1852	2278	2273	2291	2164
Specific gravity *ρ_cr_* [kg/m^3^]		2603	2675	2711	2711	2647
Absorption capacity by weight *n_w_* [%]		8.3	3.8	3.8	3.5	4.8
Open porosity *ϕ_p.op_* [%]		15.3	8.6	8.6	8.0	10.3
Total porosity *ϕ_p.tot_* [%]		28.9	14.8	16.1	15.5	18.3
Tightness *S* [%]		75.0	81.5	85.2	83.9	83.5
Sand (HCl-insoluble part) content	*C_agg_*_,*s*%_ [%]	57.1	24.2	25.8	29.7	34.1
	*C_agg_*_,*s*_ [kg/m^3^]	1057.5	551.3	586.5	680.3	737.9
Limestone aggregate content	*C_agg_*_,*d*%_ [%]	13.6	56.3	56.2	51.5	45.5
	*C_agg_*_,*d*_ kg/m^3^]	251.9	1282.7	1277.6	1179.6	984.5
Aggregate content	*C_agg_*_,%_ [%]	70.7	80.5	82.0	81.2	79.6
	*C_agg_* [kg/m^3^]	1309.4	1834.0	1864.2	1859.9	1722.4
Attached components content	*C_att_*_,%_ [%]	6.3	6.3	4.9	5.4	5.4
	*C_att_* [kg/m^3^]	116.7	143.5	111.4	123.7	116.8
Cement content	*C_cem_*_,%_ [%]	23.0	13.2	13.1	13.4	15
	*C_cem_* [kg/m^3^]	426.0	300.7	297.8	306.9	324.6

**Table 6:** Test and calculation results for samples D.1÷D.4 and averaged sample D.5, taken at distance of 0.30 m from top of ETG-1 pole.

Parameter		Samples				
		D.1	D.2	D.3	D.4	D.5
Apparent density *ρ_c_* [kg/m^3^]		2268	2297	2277	2220	2257
Specific gravity *ρ_cr_* [kg/m^3^]		2715	2706	2667	2688	2682
Absorption capacity by weight *n_w_* [%]		3.8	3.5	3.6	4.0	3.7
Open porosity *ϕ_p.op_* [%]		8.7	8.0	8.3	9.0	8.3
Total porosity *ϕ_p.tot_* [%]		16.5	15.1	14.6	17.4	15.8
Tightness *S* [%]		83.5	84.9	85.4	82.6	84.2
Sand (HCl-insoluble part) content	*C_agg_*_,*s*%_ [%]	25.7	27.3	28.0	27.8	29.7
	*C_agg_*_,*s*_ [kg/m^3^]	582.9	627.1	637.5	617.3	670.5
Limestone aggregate content	*C_agg_*_,*d*%_ [%]	56.4	54.4	53.3	53.3	50.2
	*C_agg_*_,*d*_ kg/m^3^]	1279.1	1249.6	1213.6	1183.5	1133.2
Aggregate content	*C_agg_*_,%_ [%]	82.1	81.7	81.3	81.1	79.9
	*C_agg_* [kg/m^3^]	1862.0	1876.8	1851.1	1800.8	1803.7
Attached components content	*C_att_*_,%_ [%]	5.0	5.2	5.5	5.3	5.3
	*C_att_* [kg/m^3^]	113.4	119.5	125.2	117.7	119.6
Cement content	*C_cem_*_,%_ [%]	12.9	13.1	13.2	13.6	14.8
	*C_cem_* [kg/m^3^]	292.6	300.9	300.5	302.0	334.1

## Data Availability

The original contributions presented in this study are included in the article material. Further inquiries can be directed to the author.
